# An automated liquid jet for fluorescence dosimetry and microsecond radiolytic labeling of proteins

**DOI:** 10.1038/s42003-022-03775-1

**Published:** 2022-08-25

**Authors:** Matthew Rosi, Brandon Russell, Line G. Kristensen, Erik R. Farquhar, Rohit Jain, Donald Abel, Michael Sullivan, Shawn M. Costello, Maria Agustina Dominguez-Martin, Yan Chen, Susan Marqusee, Christopher J. Petzold, Cheryl A. Kerfeld, Daniel P. DePonte, Farid Farahmand, Sayan Gupta, Corie Y. Ralston

**Affiliations:** 1 Sonoma State University, Rohnert Park, Sonoma, CA 94928 US; 2 Molecular Biophysics and Integrated Bioimaging Division, Lawrence Berkeley National Laboratory, Berkeley, CA 94720 US; 3 Center for Synchrotron Biosciences, School of Medicine, Case Western Reserve University, Cleveland, OH 44106 US; 4 Biophysics Graduate Program, University of California, Berkeley, CA USA; 5 MSU-DOE Plant Research Laboratory and Department of Biochemistry and Molecular Biology, Michigan State University, East Lansing, MI 48824 US; 6 Environmental Genomics and Systems Biology Division, Lawrence Berkeley National Laboratory, Berkeley, CA 94720 US; 7 Biological Systems and Engineering Division, Lawrence Berkeley National Laboratory, Berkeley, CA 94720 US; 8 Department of Molecular and Cell Biology, University of California, Berkeley, CA USA; 9 Department of Chemistry, University of California, Berkeley, CA USA; 10 California Institute for Quantitative Biosciences, University of California, Berkeley, CA USA; 11 Stanford Linear Accelerator Center, Menlo Park, CA 94025 US; 12 Molecular Foundry Division, Lawrence Berkeley National Laboratory, Berkeley, CA 94720 US

**Keywords:** Biological techniques, Biophysical methods, Structural biology

## Abstract

X-ray radiolytic labeling uses broadband X-rays for in situ hydroxyl radical labeling to map protein interactions and conformation. High flux density beams are essential to overcome radical scavengers. However, conventional sample delivery environments, such as capillary flow, limit the use of a fully unattenuated focused broadband beam. An alternative is to use a liquid jet, and we have previously demonstrated that use of this form of sample delivery can increase labeling by tenfold at an unfocused X-ray source. Here we report the first use of a liquid jet for automated inline quantitative fluorescence dosage characterization and sample exposure at a high flux density microfocused synchrotron beamline. Our approach enables exposure times in single-digit microseconds while retaining a high level of side-chain labeling. This development significantly boosts the method’s overall effectiveness and efficiency, generates high-quality data, and opens up the arena for high throughput and ultrafast time-resolved in situ hydroxyl radical labeling.

## Abstract (web summary)

A high-speed liquid jet delivery system improves the X-ray footprinting and mass spectrometry method to label proteins for structural studies.

## Introduction

X-ray radiolytic labeling, also known as X-ray footprinting and mass spectrometry (XFMS), is an in situ hydroxyl radical (•OH) labeling method in which X-ray irradiation dissociates water to produce hydroxyl radicals that covalently modify solvent accessible amino acid side chains^1,2^. In regions where a protein is folded or bound to a partner, side chains are inaccessible to solvent, and therefore protected from labeling. The subsequent analysis via liquid chromatography coupled with mass spectrometry provides both identification and quantification of those covalent modifications at the single residue level^3,4^. XFMS is not limited by specialized sample preparation requirements and enables the investigation of various functional states of dilute proteins in the solution state, yielding information that is often unattainable by high-resolution methods such as X-ray crystallography, nuclear magnetic resonance (NMR), and Cryogenic electron microscopy (CryoEM). Other non-radiolytic •OH labeling methods such as Fenton chemistry^5–8^ or UV-laser-induced •OH labeling^9,10^ provide solvent accessibility information similar to XFMS, and are excellent methods in the absence of a synchrotron facility. However, such non-radiolytic methods require the addition of H_2_O_2_, which can cause uncontrolled conformational changes or oxidations^11,12^. In XFMS, low energy transfer radiation can produce •OH isotropically wherever there is water, both in bulk and within the protein interior. Due to its high reactivity, the •OH molecule does not diffuse >5 Å before covalently modifying nearby residue side-chains, interacting with buffer, or undergoing self-recombination^1,3^. Protein modifications, therefore, occur at unique residues for which a water molecule was either in close proximity or hydrogen-bonded to the protein backbone or side chain. This effectively provides a “water map”: a location of the water molecules surrounding and within the protein at the time of irradiation. As most conformational hot spots are associated with water networks with conserved residues, XFMS has been proven very useful when comparing different states of proteins to determine local structural changes such as during folding, binding, activation, or aggregation^13–16^.

We implemented microsecond XFMS at the Advanced Light Source (ALS) at Berkeley Laboratory in 2012;^1^ the method had previously been pioneered in the late nineties at the National Synchrotron Light Source (NSLS) at Brookhaven National Laboratory and continues to serve the structural biology community to date at the NSLS-II^17–19^. The use of synchrotron X-rays enables an extremely high photon flux density, translating into a high steady-state concentration of •OH, which in turn enables very short exposures for generating an adequate yield of detectable modification products. It is important with XFMS data collection to achieve the shortest possible exposure to minimize secondary radiation damage, while still varying the integrated X-ray dose 2–20-fold in order to generate a hydroxyl radical dose–response range large enough for accurate quantification of the hydroxyl radical modification rates. Maintaining these two factors as the prime drivers for development, the XFMS method has continually evolved for the past several decades at synchrotron X-ray facilities. However, the standard capillary flow method for XFMS poses several challenges: the glass of the capillary consumes a significant portion of the X-ray dose, the high energy beam degrades the capillary over time, and the heat deposited in the capillary causes uneven flow, clogging, and ultimately breaking of the capillary. Recently, we developed a microfluidic containerless approach that increases •OH labeling efficiency by 10-fold in protein samples, and demonstrated that this approach can be used with an unfocused broadband X-ray beam to generate useful XFMS data on the hundreds of microsecond timescale^20^.

Here we report on two significant advances to the XFMS experiment that leverage the microfluidic liquid jet sample delivery system. First, we implemented the liquid jet system at a focused broadband X-ray beamline, with increased flux density as high as 100-fold compared to an unfocused beam, as estimated from the difference in Alexa degradation rate measurements^18^^,^^21^. This allowed exposures as short as one microsecond and enabled the use of highly •OH scavenging buffers such as tris(hydroxymethyl)aminomethane (Tris) in the XFMS experiment. Second, we implemented an automated inline fluorescence X-ray dose analysis system. This system records fluorescence from the moving liquid immediately after X-ray irradiation and before capture in the collection tube containing a quenching agent. Using automated LabVIEW-based analysis of Alexa fluorescence emission, we demonstrated that dosage measurements can now be completed rapidly inline immediately subsequent to protein irradiation. A detailed description of instrument design, timing, and automation are described in Supplementary Notes 1 and 2, and Supplementary Figs. S1 through S16. These advances represent a significant improvement over the current standalone manual fluorometer mode of dosage measurement, which is error-prone and time-consuming. We further characterized the instrumentation using single-digit microsecond exposures to obtain high-yield hydroxyl radical modification products on both cytochrome c (cyt c) and the megaDalton phycobilisome (PBS) light-harvesting antenna protein complex.

Together, the software and hardware instrumentation significantly advance the throughput and ease of use of the XFMS experiment for protein structural studies. In addition, the single-digit exposure regime now opens up an opportunity for microsecond pump-probe and time-resolved XFMS experiments.

## Results

### Design and operation of inline automated Alexa dose–response analysis

**Fig. 1:**
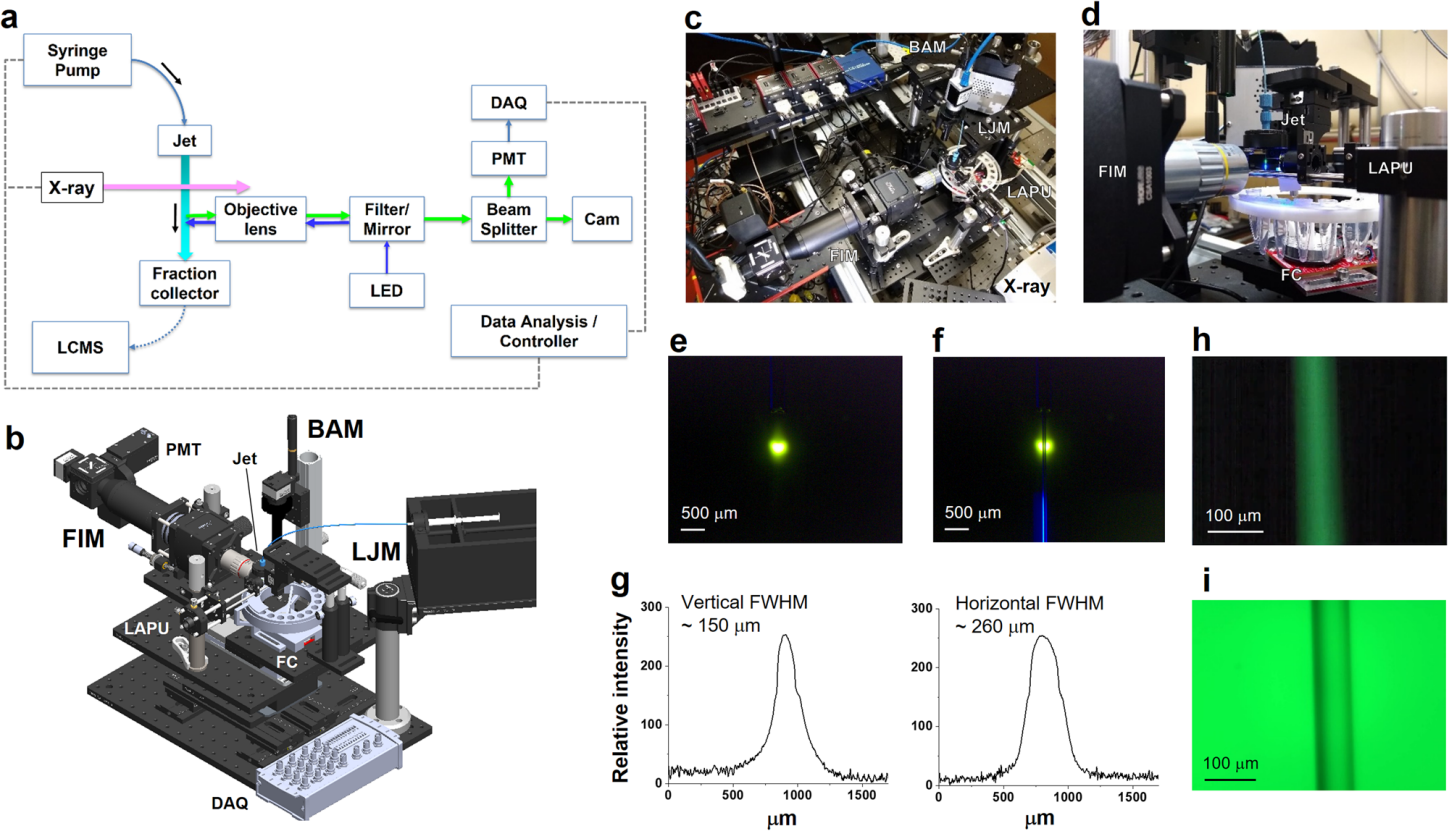
Instrument design for automated liquid-jet fluorescence dosimetry and sample exposure. **a** Block diagram showing system architecture of integrated components operated by a LabVIEW-based controller. The critical components include: high-pressure pump, polished microcapillary jet nozzle, fraction collector (FC), wavelength-specific LED, filters (excitation and emission), dichroic mirror, objective lens, a photomultiplier tube (PMT), data acquisition (DAQ), and imaging camera (Cam). **b** Computer-aided design of the physical setup at the beamline showing critical modules and components–beam alignment module (BAM), liquid jet module (LJM), fluorescence imaging module (FIM), and laser assisted pre-alignment unit (LAPU). **c** Top view of the instrument assembly, which is under operation at the beamline 5.3.1 at the Advanced Light Source. Original photograph taken by author S. Gupta. **d** Side view of the instrument showing how the LAPU assists in focusing the microfocused X-rays on the sample before the Alexa fluorescence data collection by the FIM. **e**, **f** Position of microfocused X-ray beam, and fluorescence excitation on the path of a 75 μm liquid jet captured by the BAM at low magnification. Original photograph taken by author S. Gupta. **g** X-ray beam size was determined from the image analysis using ImageJ (https://imagej.nih.gov/ij). **h** Camera inline view of fluorescence emitted from the 75 μm sample jet capture by the FIM during automated Alexa DR analysis (Fig. 3). **i** Camera inline view of a visible image of 75 μm sample jet captured by the FIM during sample exposure.

Proteins are intrinsic •OH scavengers, and buffers or additives can be extrinsic •OH scavengers. The amount of scavenging therefore can vary widely from sample to sample, affecting the yield of •OH modifications products. X-ray dose measurements using Alexa fluorescence at 520 nm, in which the rate of fluorescence bleaching provides an empirical estimate of effective •OH dose to the protein, is well-established and widely used in the XFMS method for optimizing protein sample exposures and normalizing sample to sample dose variation^13,18^. Currently, this dose optimization is carried out as a separate experiment, either using a plate reader or a standalone spectrometer separate from the sample exposure setup. Here we report on the development of real-time and inline Alexa fluorescence analysis for XFMS using a microfluidic liquid jet. In our previous report, we demonstrated the successful use of a microfluidic liquid jet to obtain a tenfold increase in modification level compared to using a conventional capillary flow method for protein samples at an unfocused broadband synchrotron beamline at the ALS^20^. When the liquid is ejected from the nozzle, the shape of the liquid is dependent on several factors such as viscosity, surface tension, and flow velocity. We have shown that under the same solution conditions, the liquid jet’s performance and stability depend on the flow velocity and nozzle diameter. There is an upper and lower flow velocity beyond which the liquid jet breaks up and is not suitable for XFMS^20^. The smaller the diameter of the jet, the wider the jetting regime, because a narrow jet can be operated with a wider range of sample speeds. As a result, a narrow jet will provide more options for exposure times, which will directly benefit the dose–response analysis by providing a wider range of deliverable hydroxyl radical dose (Supplementary Fig. S17). However, the length of the stable jet stream decreases with nozzle diameter. We observed that a 50–100 μm diameter nozzle could produce a stable jet 1–3 cm long, which provides opportunities to develop high sensitivity containerless optical probes of the sample during its transit from the jet nozzle to the collection tube. This concept is based on the well-known microfluidics chip-based light-induced fluorescence (LIF) configuration. The instrument’s layout for automated XFMS sample exposure and Alexa dose–response analysis is shown in Fig. 1a–d.

The instrument contains three main modules: the liquid-jet module (LJM), a beam alignment module (BAM), and a fluorescence imaging module (FIM). The LJM includes the liquid jet delivery and sample collection stage. A high-pressure syringe pump generates a high flow velocity through the interchangeable nozzle with a diameter as narrow as 50 μm. The pump and jet nozzle can operate with high precision to deliver a stable liquid stream with a volume <20 μl per injection at a flow speed of 2–50 m/s. The microfluidic flow path between the pump and nozzle is kept at a minimum to reduce the backpressure, and the samples are collected in a fraction collector. The liquid jet is aligned with the microfocused X-ray beam in such a way that the X-rays expose the vertically oriented liquid jet homogenously (Fig. 1d–f). For a given nozzle diameter, the exposure time is determined by the full-width half maximum (FWHM) of the beam’s vertical height (Fig. 1g). The BAM is a high precision alignment module which combines captured X-ray fluorescence from a diamond or Nd YAG screen, jet edge-illumination using total internal reflection properties of the liquid column, and high-resolution image recognition with a center detection algorithm using Python-based control and analysis software (Fig. 1f and Supplementary Note 1). This module also has a laser-assisted pre-alignment unit (LAPU) that helps to bring the micron size focused beam within <700 μm of the jet stream (Fig. 1d and Supplementary Note 2). The FIM applies the principle of LIF for microfluidics. In this design, collimated blue light from an LED is first passed through an excitation bandpass filter and then reflected with a dichroic mirror into an objective lens to focus the excitation light onto the liquid jet underneath the X-ray exposure region (Fig. 1d, e). The emission of Alexa fluorescence dye from the sample solution is channeled back through the same objective lens into the filter cube, where it passes through the dichroic mirror and a bandpass emission filter to cut off unwanted background wavelengths. We used 10× or 20× infinity corrected long working distance objective lenses to achieve high fluorescence signal intensity from the field of view that covers ~1 mm of the jet stream. The use of a long working distance (>30 mm) lens provided sufficient space for handling and manipulating the beam alignment components and fraction collector around the liquid jet. The emitted light from the filter cube was split into two by a beam splitter for both visualizing the jet and quantitative fluorescence measurements (Fig. 1h, i). Only 10% of the emitted light is used for quantitative fluorescence analysis using a photomultiplier tube (PMT). A millisecond optical shutter is used to collect fluorescence emission for a fractional duration of the overall runtime for sample exposure and collection. The remaining 90% of the emission is directed to a camera in order to visually monitor the liquid in real-time. Signals and images from the three modules are viewed in a LabVIEW-based GUI for control of the individual components, quantifying fluorescence emission, and calculation of the dose rate and overall automation of the XFMS sample exposure experiments. Through the LabVIEW control panel, the user can define the number of samples to expose, the volume of those samples, and the amount of X-ray exposure per sample. The timing of jetting and X-ray exposure and simultaneous collection of the fluorescence emission data is a critical part of the automation, and details on timing consideration are described in Supplementary Note 1. Our main goal was to collect reproducible fluorescence data from a high-speed liquid jet with the shortest possible exposure time and the smallest possible volume of sample. We collected reproducible fluorescence data from a high-speed liquid jet with a minimum of 2 μs exposure time while consuming only 25 μl of sample.

### Performance of inline fluorescence detection

**Fig. 2:**
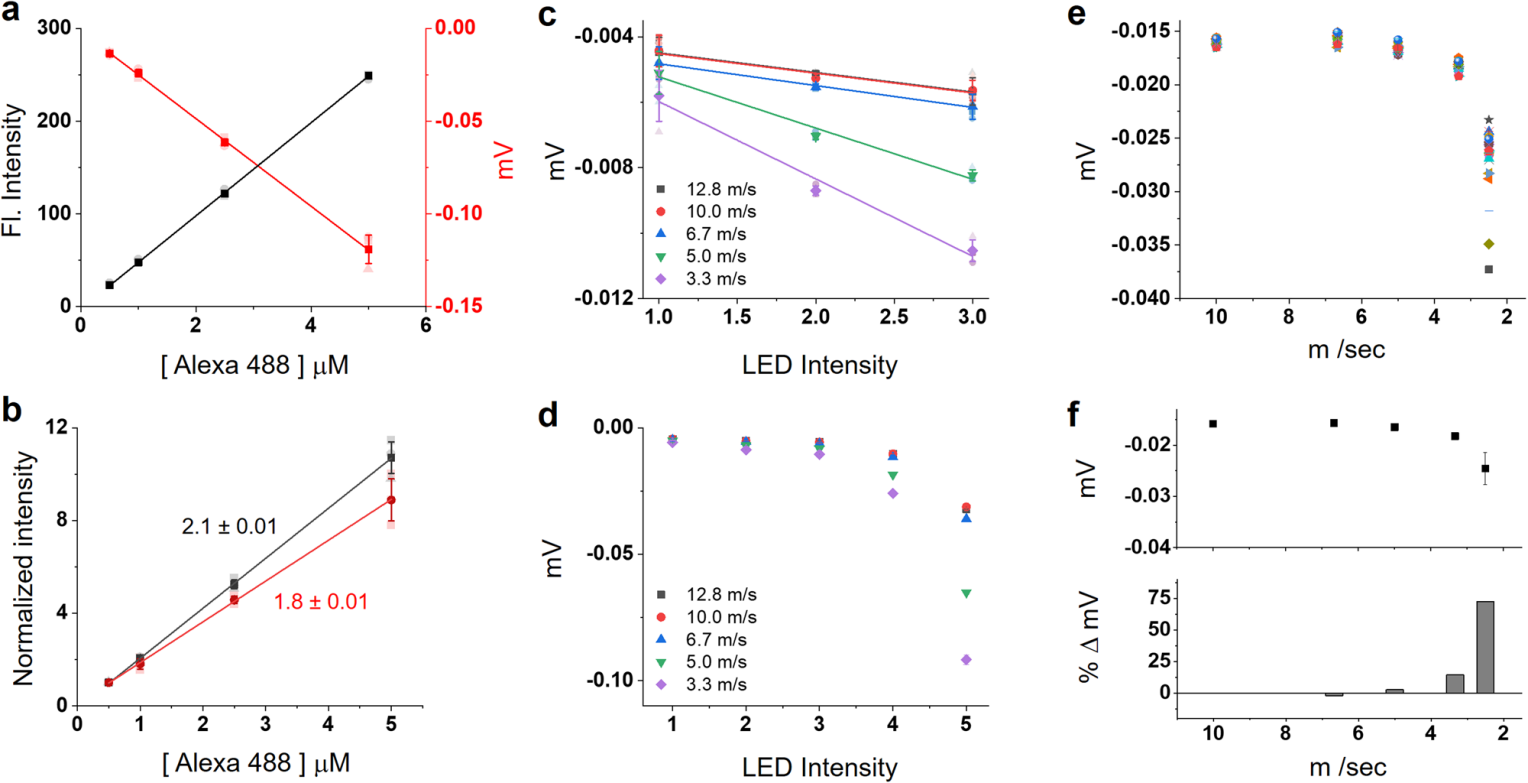
Testing and verification of data acquisition from liquid-jet fluorescence. **a** Concentration-dependent Alexa fluorescence readings obtained by a standalone spectrofluorometer (left axis, black line) are compared with PMT voltage readings from the sample jet (right axis, red line). The normalized data are shown in **b** where solid lines represent the linear fit, and slope values are indicated. **c** PMT response with an increase in excitation light intensities, which is used to vary fluorescence emission from the sample jet. Lower jet speed generates more non-linearity, as shown in **d.** **e** PMT response with the decrease in jet speed. Data was obtained from ~50 replicates. The percentage variation for each speed relative to the highest speed is shown in **f**. These values are used to process raw PMT data (Fig. 3a) and generate the final Alexa dose responses shown in Fig. 3b. All error bars represent the standard deviation of three or more repeats. Numerical source data are available in Supplementary Data 1.

To determine the accuracy of the FIM, voltage measurement data from the PMT for different concentrations of Alexa 488 dye were compared to a standalone fluorometer. The inline fluorescence was measured using a total sample volume of 25 μl of Alexa 488 at concentrations ranging from 0.5–5 µM, which are the standard concentrations used for Alexa dosimetry in an XFMS experiment. The samples were ejected as a liquid jet of 50 μm diameter at a fixed speed of 20 m/s. The emission intensities were collected from 0.9 μl volume of illuminated sample, while the data acquisition duration (integration time) was 50 ms (Supplementary Note 1). In contrast, for the manual measurements, all samples were diluted 100 times in a cuvette having a path length of 1 cm and an integration time of ~5 sec on a portable standalone spectrofluorometer. Due to the nature of the different measurement modes, the slope of the resulting intensity response plot for inline fluorescence was negative, while the slope for manual measurement was positive, though the absolute value of the linear regression is the metric of interest for accuracy comparison. The rate of decrease in fluorescence is 1.16 times faster when measured by the PMT voltage versus the standard fluorometer readings (Fig. 2a, b). This comparison provided an evaluation of our system as the commercial fluorometer is a highly accurate and calibrated instrument used as the industry standard for the quantification of fluorescence. We also evaluated the overall repeatability of the system under various LED excitation intensities and the influence of various jet speeds (Fig. 2c–e). Generally, each LED intensity showed a high level of repeatability apart from the lowest setting as indicated by the error bars in Fig. 2c. It is important to note that when the FIM is focused on the jet at its highest speed, the non-linearity in each set of speed responses is due to a combination of the slight positional shift of the jet and its impact on the depth of field of microscopic imaging (Fig. 2d). Such an effect was prominent only for the low-speed ranges, in which the PMT values often showed a percentage variation higher than >20% (Fig. 2f). The data processing for fluorescence dose–response analysis, which is automatically analyzed after each set of samples, is described in Supplementary Note 1.

### Inline fluorescence analyses of a buffer and protein solution

**Fig. 3:**
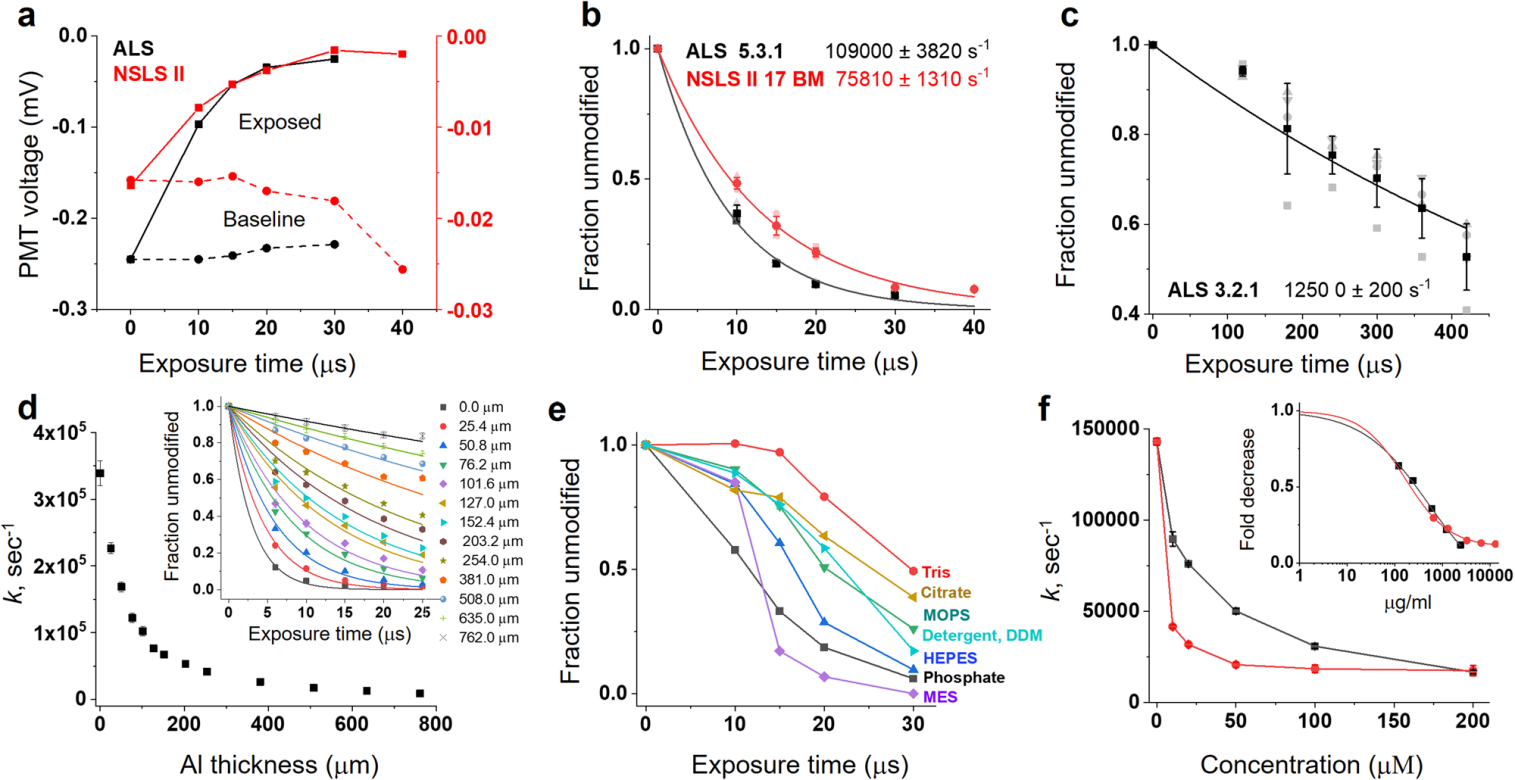
Automated Alexa dose–response analysis of buffer and protein samples. **a** Raw PMT voltage data of Alexa dose–response at NSLS-II 17-BM and ALS 5.3.1 beamlines running at 80% of maximum beam current. **b** Automatically processed Alexa dose–response plot in phosphate buffer at focused X-ray beamline NSLS-II 17-BM and ALS 5.3.1 running at 80% of maximum beam current. Solid lines represent single exponential fits. **c** Automatically processed Alexa dose–response plot in phosphate buffer at unfocused X-ray beamline ALS 3.2.1. Solid line represents a single exponential fit. **d** Automatically processed Alexa dose–response plots (inset, solid lines represent single exponential fits) to determine reactivity rate under different degrees of beam attenuation by Aluminum. The data was collected at ALS 5.3.1 running at 100% of maximum beam current. **e** Alexa dose–response of biological buffers and DDM with varying degrees of intrinsic •OH scavenging properties. Solid lines interconnect the data points. **f** Effect of protein concentration on Alexa hydroxyl reactivity rate measured by automated dose–response for cyt c (~12 kDa, red) and BSA (~65 kDa, black). Solid lines interconnect the data points. Inset shows the normalized data with sigmoidal fit and provides the trends in the protein’s intrinsic •OH scavenging depending on total protein content. All error bars represent the standard deviation of three or more repeats. Numerical source data are available in Supplementary Data 1.

To test the device performance mimicking actual sample exposure conditions, we performed Alexa fluorescence assays of various buffers and cyt c solutions. The liquid jet was aligned to the microfocused X-ray beam at ALS beamline 5.3.1 and NSLS-II beamline 17-BM. A 100–120 μm vertical height (FWHM) of the beam and 50 μm to 100 μm jet diameters provided single-digit microsecond exposures using sample speeds that fit well within the jetting regime (Supplementary Table 1). At the same time, the high-speed created a sufficiently structurally stable jet for post-exposure inline spectroscopy^20^. The excitation/emission probe from the FIM was positioned ~2–5 mm beneath the sample exposure window to monitor the changes in Alexa fluorescence resulting from X-ray-induced degradation or photobleaching. The fluorescence emission was collected using the same sample volume and timing configuration as described in the previous section and Supplementary Note 1. Figure 3a, b shows the raw and the processed data for Alexa fluorescence degradation in phosphate buffer with the increase in the exposure time (or decrease in jet speed) at ALS beamline 5.3.1 and NSLS-II beamline 17-BM. To correct for the effect of jet speed and depth of field on the PMT output, we collected Alexa fluorescence readings from each of the speeds without enabling the X-ray beam, and the readout was used for baseline correction as described in Supplementary Note 1. We obtained a full dose–response using exposure ranges in the tens of microseconds, representing the shortest exposure times reported to date for XFMS experiments using a high flux density beamline. We also carried out automated dose–response studies at the low flux density unfocused ALS beamline 3.2.1 (Fig. 3c). We obtained exposure ranges in the hundreds of microseconds, which corroborated the data in our previous report using a manual sample exposure setup at a low flux density beamline^20^. All Alexa 488 hydroxyl radical dose responses produced by our automated instrumentation were reproducible with manual measurements using a standalone fluorescence spectrometer, as shown in Supplementary Fig. S18. We further tested the sensitivity and usefulness of the automated system in analyzing loss of dose by beam attenuation, strongly scavenging buffers, and the increasing amount of protein content in the sample. Automated Alexa dose responses distinguished and determined the hydroxyl radical reactivity rates for various degrees of X-ray attenuation by aluminum (Fig. 3d and Supplementary Table 2). Although detailed calculations of the effect of aluminum attenuation on the absorbed flux by the sample is beyond the scope of our current studies, the plot shown in Supplementary Fig. S19 provides calculated flux spectra for various thickness of aluminum at beamline 5.3.1. Our results show the sensitivity of the instrument for determining Alexa reactivity from high-quality dose–response profiles obtained by fine-tuning the X-ray dose.

The method was applied to various buffer solutions with intrinsic •OH-scavenging properties, which generated Alexa dose–response profiles as expected from the previous reports^18^. The noticeable lag phases or the slower decay of Alexa in Tris, HEPES, and MOPS are due to the hydroxyl radical scavenging of these buffer constituents, which have higher intrinsic reactivities for •OH compared to phosphate buffer at similar concentrations, and which are present at 1000 fold higher concentration than Alexa 488 (Fig. 3e)^22^. Therefore, prolonged exposure is needed to build up a higher steady-state hydroxyl radical dose that will compensate for the loss of •OH by competitive scavenging reactions with buffer. Protein concentration-dependent Alexa dose analysis with BSA (~65 kDa) and cyt c (~12 kDa) showed expected results, in which a decrease in the •OH reactivity of Alexa coincides with an increase in the protein concentration and molecular weight (Fig. 3f). The inline fluorescence analysis provided qualitative information about the relationship between protein content and hydroxyl radical reactivity. This type of rapid Alexa dose data is of great value when running XFMS experiments with previously untested samples which vary in overall protein content (inset Fig. 3f). To test the effect of quenching, manual fluorescence measurements were taken after irradiation with a high flux density beam, and no quenching buffer was used. In this case, the dose–response kinetic traces presented an apparent insensitivity to buffer and protein scavenging properties (Supplementary Fig. S20). This is likely because secondary free radicals can degrade Alexa 488 fluorescence during the time it takes for post-exposure sample handling; essentially all secondary damage is complete by the time the methionine amide is added. It is noteworthy that adding methionine amide to quench unwanted secondary damage to samples exposed using the mechanical shutter and high-throughput 96 well plate mode takes several minutes, presenting a significant risk of incorporating post-exposure secondary radial mediated protein modification and Alexa damage. In contrast, the liquid jet sample is collected directly into the tube with methionine amide, and the quenching step is almost instantaneous. In addition to the significant improvement in time efficiency, the automated inline fluorescence method gives an advantage over conventional standalone fluorescence measurement by increasing the sensitivity of detection of hydroxyl radical scavengers, and providing a more accurate measure of scavenging conditions.

### Single-digit microsecond exposure yields modification in cyt c

**Fig. 4:**
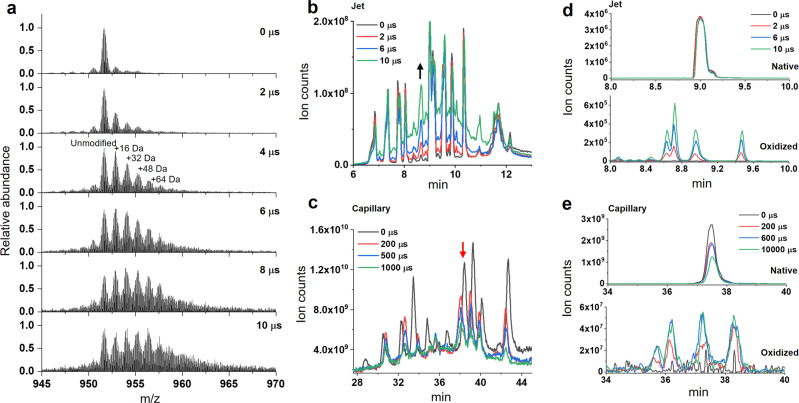
High-dose single-digit microsecond exposure of a protein sample. **a** Intact mass analysis of exposed cyt c shows a significant amount of modification within 10 μs. **b** Total ion chromatogram of exposed and trypsin digested cyt c using a 50 μm liquid jet and single-digit microsecond exposure. The arrow indicates the progressive increase in modified peptides with increasing exposure time. **c** Total ion chromatogram of exposed and trypsin digested cyt c using 200 μm ID capillary using a standard flow setup, which used 150 μm Al attenuation to prevent sample damage and glass capillary breakage. The arrow indicates the significant decrease in unmodified peptides with increasing exposure time. **d** Extracted ion chromatogram of the unmodified (390.21 m/z, z = 3) and +16 Da modified (395.54 m/z, *z* = 3) cyt c peptide ^28^TGPNLHGLFGR^38^ using 50 μm liquid jet and single-digit microsecond exposure. **e** Extracted ion chromatogram of the same peptide as that in D, using 200 μm ID capillary in the standard flow setup with 150 μm Al attenuation. Numerical source data are available in Supplementary Data 2.

**Fig. 5:**
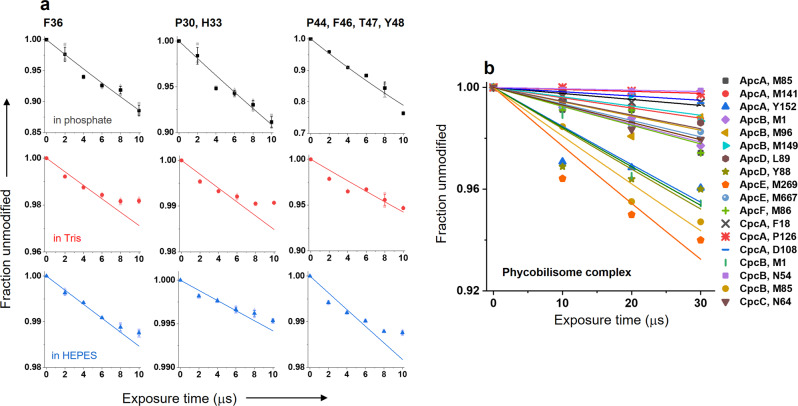
Site specific dose–response of protein samples. **a** Comparison of residue-specific dose responses of cyt c in various buffers. All error bar represent standard deviation of three repeats. Dose–response plots for the full range of residues analyzed in this study are shown in figure S5. **b** Residue-specific dose–response of the >6 megaDa PBS complex. The solid lines in all the plots represent single exponential fit (see methods section) with the *R* values ranging from 0.98–0.99. Numerical source data are available in Supplementary Data 1.

We used cytochrome *c*, a well-known standard for the XFMS method, for quantitative analysis of the effectiveness and overall performance of the liquid jet setup with the unattenuated microfocused extremely high flux density X-ray beam at the NSLS-II beamline 17-BM^17^. Such an X-ray beam causes substantial radiation damage and is impossible to use without attenuation with standard sample delivery methods such as a high-throughput 96 well plate-electronic shutter assembly or conventional microcapillary flow^23^. We tested cyt c samples in three types of buffers - phosphate, Tris, and HEPES—and used exposure times ranging from 2 μs to 40 μs with a minimum sample consumption of 25 μl for each exposure. The protein’s intact mass analysis shows a significant and progressive increase in the amount of side-chain hydroxyl modification from 2 to 10 μs X-ray exposure in phosphate buffer (Fig. 4a). The LCMS ion chromatogram of the trypsin digested cyt c exposed in the standard capillary instrument, which is only feasible with exposure time starting from 200 μs and 150 μm of aluminum attenuation, showed a significant sample loss with an increase in exposure time. Such sample damage is indicated by the reduction in peak intensities of several peptides throughout the chromatogram (Fig. 4c). In contrast, the sample exposed in the liquid jet instrument retained the integrity of the overall chromatograph better towards the higher end of X-ray dose or exposure (Fig. 4b). For a few peptides, the high yield of modifications was clearly indicated by an increase in the abundance of the modified peak with the rise in the exposure time. The difference is evident when we compared relative abundances of modified and unmodified peptides between the liquid jet and capillary setup (Fig. 4d, e). The X-ray dose and exposure conditions should only affect the relative level of unmodified vs. modified peptide fragments. The relative levels should not be significantly altered by the same digestion and standard liquid chromatography coupled to the electrospray ionization method used for XFMS analysis of the same protein preparations. A comparison of the extracted ion chromatogram showed that the disappearance of the unmodified peak is much higher than that of an increase in the abundance of modification. Recent reports showed heat-induced capillary damage^20^ and fragmentation because of radiation damage under prolonged exposure when using the capillary method^24^. Exposed samples analyzed by standard bottom-up LCMS showed up to 30% modification within a 10 μs exposure range in phosphate buffer (Fig. 5a and Supplementary Fig S21). For example, in many cases, for residue Phe 36 and residues Pro 44, Phe 46, Thr 47, and Tyr 48, the observed extent of change in fraction unmodified was higher than previously reported by standard modes of sample exposure^15,17^. The linearity of the pseudo-first-order dose–response traces in phosphate buffer indicated no significant secondary radiation damage within the selected dose range. The abundant chromatography peaks resulted from the minimization of sample damage, and the larger yield of side-chain modification provided better MS/MS mass assignments to identify modification sites. This represents a significant improvement in data quality that can be obtained in the XFMS method. The modification yield with Tris and HEPES was relatively low compared to that of the phosphate buffer, as expected from their scavenging properties. Cyt c in Tris and HEPES buffer showed a larger extent of change in the fraction of modification and better linearity in the dose–response when the exposure range was extended from 8 to 25 μs (Supplementary Fig. S21). Previously, many studies have shown that a low level of exposure does not affect footprinting analysis as long as the dose–response is linear within statistical significance^13,15,25,26^. Further, in the XFMS method in general, exposure times are kept short to limit modification to a few percent in order to prevent any effect of modification on protein structure^2^. The modification levels (~1–2%) we report here for Tris and HEPES show that using the liquid jet delivery method at a high flux density microfocused beamline, it is now possible to carry out XFMS studies in those buffers (Fig. 5a and Supplementary Fig. S21). Overall, the liquid jet sample exposure setup delivered short doses, reduced secondary radiation damage, consumed less sample, and generated high-quality data.

### Prospects for studying large complexes

XFMS provides an alternative and highly complementary structural approach for the study of large protein complexes compared to the more well-known biophysical and structural biology tools such as crystallography, CryoEM, and NMR. While recent advances in the XFMS instrumentation have made the approach much more tractable for large complexes than before, large proteins or protein assemblies are still challenging because they effectively act as strong intrinsic •OH scavengers, in the sense that a higher •OH concentration is required to produce the same level of oxidation as compared to smaller proteins. In addition, large complexes often require the presence of a high concentration of extrinsic •OH scavengers to maintain structural integrity, such as cofactors, detergents, and phospholipids. A lack of adequate photon flux density requires longer exposure times for delivery of a sufficient dose, which increases secondary radiation damage, resulting in misleading dose–response kinetics due to protein unfolding or radiation damage. XFMS studies on several membrane proteins were only possible at the NSLS X28C footprinting beamline after installing a focusing mirror, which increased the usable flux density by over an order of magnitude^21^. The focused beam capability at the NSLS-II on beamline 17-BM, and currently planned at the ALS on beamline 3.3.1^27^, is designed to provide even higher flux densities, and the instrumentation described in this report enables the full use of the high flux density beams at these facilities. We tested the XFMS instrumentation at the NSLS-II beamline using the challenging > 6 megaDalton PBS complex (Fig. 5b). The PBS is a water-soluble light-harvesting antennae complex in cyanobacteria, and interacts with the Orange Carotenoid Protein (OCP) as well as the photochemical reaction centers embedded in membranes^28–30^. Previously, we made significant progress in understanding this photoregulation mechanism in photosynthetic organisms utilizing the suite of methods of crystallography, small-angle scattering, hydrogen-deuterium exchange, and XFMS, and we elucidated some of the structural changes occurring within the OCP during high light conditions that allow binding with the PBS^16,26,31,32^. With our liquid jet instrumentation described in this report, we were able to determine the dose responses for sites deeply buried inside the core subunits of the PBS. This now opens up the possibility of delineating structural changes within the PBS when OCP binds. In summary, we were able to firmly establish and validate the high-dose containerless method to produce high-quality XFMS data on very large protein complexes.

## Discussion

High-quality data collection in the XFMS method entails rich MS/MS assignments, clear chromatography peak area quantification, and linear dose–response plots in order to accurately quantify hydroxyl radical reactivity rates. Despite significant improvements in high-resolution and high sensitivity LCMS systems in the last decade, quantitative identification of oxidative modifications given the low modification levels generated using the conventional glass capillary flow system has posed a major challenge for further improvement of this method with complex samples. The instrumentation described here features a high-speed liquid jet delivery system that utilizes the full potential of a high flux density microfocused X-ray beam, resulting in the elimination of unwanted secondary radical interactions and a significant increase in site-specific modifications, as demonstrated for both small globular proteins and a megaDalton protein complex. Recently, integrated inline OH radical dosimetry has been introduced to a UV-laser-induced •OH labeling method^33,34^. Our integrated instrument for an accurate fluorescence hydroxyl radical dose–response analysis inline with the liquid jet is a major improvement in the field of XFMS. It also significantly cuts the sample consumption to a minimum of 25 µl sample volume per exposure and allows for X-ray exposures as low as 2 µs. Consumption of sample and exposure time could be further reduced by using a gas dynamic virtual nozzle^35,36^. In contrast to current Alexa dose–response methods using single tubes or well plates, the inline fluorescence measurements determine the effect of scavengers more accurately because there is minimal time (~microseconds) between X-ray irradiation and fluorescence measurements. As part of the instrumentation, we also developed a fast and automated method to ensure precision alignment of the liquid jet sample to the X-ray source; this technology represents a straightforward approach that can now advance liquid jet-based instrumentation used in other X-ray applications at XFEL and synchrotron facilities^37–39^. Together, these contributions yield a more than 100-fold reduction in experimental time. Previously, collection of a full Alexa dose–response dataset required up to two hours of time, whereas now with inline fluorescence measurements, the same data can be collected in minutes. With the addition of the automated sample collection, a protein exposure dataset can be completed within seconds. These advances also minimize sample volume, drastically reducing the cost of the experiment. Further, the flow path can readily accommodate a mixing setup for time-resolved XFMS as a future capability. The high flow rate of a liquid jet enables two samples to mix efficiently inside a microfabricated ultrafast mixing cell, and the delay time between mixing and X-ray exposure can be controlled by adjusting either the distance of the X-ray beam from the mixing chamber or the flow velocity of the sample. With this setup it is possible to get mixing times on the order of hundreds of microseconds before ejecting the mixed sample from the jet nozzle; this is 100-fold faster than when using the existing setup, which supports mixing on the order of milliseconds. When sample speed is used for controlling the mixing time, the •OH dose can be further controlled by changing the flux density of the incident beam. This design has considerable flexibility in selecting mixing delays and enables the use of low quantities of samples. The liquid jet approach also opens up an opportunity to synergistically combine orthogonal biophysical methods such as optical pump-probe and spectroscopy with XFMS. Fluorescence can provide information on changes in size, shape, flexibility, and conformation, as well as knowledge about the proximity of biding partners^40–44^, whereas Raman can fingerprint the changes associated with secondary structure and protein-pigment interactions^45–50^. These photoluminescence spectroscopy methods are non-invasive, highly sensitive, and adaptable to the microfluidic configuration of XFMS, and therefore most appropriate to complement solvent accessibility data. This hybrid method can provide maximum insight into the structure and kinetics of protein complexes in an integrated structural mapping approach.

## Methods

### Sample preparation and exposure

The 0.5–5 μM Alexa 488 fluorescence (Thermo Fisher Scientific) and 10–100 μM horse heart cytochrome *c* (cyt c) (Sigma) were prepared in various buffers at 10 mM buffer concentration (Figs. 3 and 4). Detergent N-Docecyl-beta-D-Maltoside (DDM) concentration was 0.2%. The wild-type phycobilisome was purified^51,52^ from cyanobacterium *Synechocystis* sp. strain PCC 6803 and exposed at a concentration of 0.5 μM in 800 mM phosphate buffer. Radiolysis of samples was performed at ALS beamline 3.2.1 and 5.3.1, and NSLS-II beamline 17-BM. The ALS beamlines 3.2.1 and 5.3.1 delivered a 3–12 keV broadband X-ray beam from a bending magnet source with an unfocused and focused beam size of 4 (V, vertical) × 10 mm^2^ (H, horizontal) (using X-ray slits) and 140–200 (V) × 360–500 (H) μm^2^ respectively. The NSLS-II 17-BM beamline is a focused 4.5–16 keV broadband 3 pole wiggler source with an adjustable beam spot size ranging from 100 (V) × 450 (H) μm^2^ to 2.6 × 2.6 mm^2^; most experiments reported here were conducted at the minimum spot size. To achieve a sample velocity within the stable jetting regime and adequate flux density, the desired beam sizes are obtained either by using X-ray slits or adjusting the mirror bend parameters in the unfocused and focused X-ray beamlines, respectively. The jet module was constructed with optomechanical components from ThorLabs in which a motorized stage held the three-axis manual translation stage with an optical disk mounted above the X-ray beam path. A tapered hole on the optical disk plate held the microtight ZDV (zero-dead-volume, 1/16” to 360 μm OD tubing connector, Idex Health & Science) adapter, in which the 50 or 100 μm diameter nozzle (made from Molex 360 OD polymicro fused silica capillary, https://www.molex.com) was mounted vertically downward such that the liquid jet passes perpendicular to the X-ray beam and the sample gets collected in a fraction collector. A high-pressure syringe pump (Harvard Apparatus) with 1 or 2.5 ml gas-tight glass lure-lock syringes (Hamilton) and 1/16”–200 μm ID Peeksil tubing (Idex Health & Science) was used the drive the sample into the jet nozzle through the microtight adapter. The sample jet was exposed ~2–5 mm downstream from the end of the polished capillary nozzle (Figure S3) with exposure time ranging from 2–40 μs at the focused beamlines (ALS 5.3.1 and NSLS-II 17-BM) and up to 1 ms at the unfocused beamline (ALS 3.2.1). Except where explicitly noted in the text, exposed protein samples were collected in micro-centrifuge tubes containing methionine amide according to the standard method of post-exposure quenching^18^. The flow rate, exposure time, and sample collection volume were controlled by LabVIEW-based software (Supplementary Table 1). Inline Alexa fluorescence was carried out using the fluorescence imaging module and automated fluorescence dose–response analysis software within the same LabVIEW-based controlled interface. In addition, all samples were collected and analyzed as required by manual fluorescence spectrometry using QuantiFluor ST (Promega) or standard bottom-up LCMS^1^. Sample preparation, exposure, and analysis were done in triplicate.

### Mass spectrometry and automated workflow for data analysis

For intact protein analysis, exposed samples were diluted 25-fold with 50 mM ammonium bicarbonate. 10 µL of sample was then injected and desalted at a flow rate of 100 µL/min of buffer A (0.1% formic acid) for 4 min on a hand-packed column (Thermo Scientific POROS R2 reversed-phase resin 1112906, 1 mm ID × 2 cm, IDEX C-128). Protein was then eluted with a gradient of 10–65% buffer B (90% Acetonitrile, 0.1% Formic Acid) at a flow rate of 20 μL/min over 5 min, and then of 65–99% buffer B over 6 seconds, and then held at 99% buffer B for 3 min prior to the execution of a sawtooth washing step and equilibration at 10% buffer B. Protein was eluted directly into a Q Exactive Orbitrap Mass Spectrometer operating in positive mode (resolution 140,000, AGC target 1e6, maximum IT 50 ms, scan range 200–2000 m/z).

For site-specific analysis, the exposed samples were digested using standard methods with trypsin enzyme (Promega) overnight at 37 °C at pH 8 in 50 mM ammonium bicarbonate buffer and analyzed on an Agilent 6550 iFunnel Q-TOF mass spectrometer (Agilent Technologies, Santa Clara, CA) coupled to an Agilent 1290 LC system (Agilent) as described previously^1,16^. The unmodified and modified peptide fragments were identified by Mascot database search of the tandem mass spectrometry data collected in the data-dependent mode. The abundance (peak area) of the identified unmodified and modified peptides at each irradiation time point area were measured from their respective extracted ion chromatogram of the mass spectrometry data collected in the precursor ion mode using Agilent Mass Hunter V 2.2 software. The fraction unmodified for each peptide was calculated as the ratio of the integrated peak area of the unmodified peptide to the sum of integrated peak areas from the modified and unmodified peptides. The dose–response curves (fraction unmodified vs. X-ray exposure) were fitted to single exponential functions in Origin® Version 7.5 (OriginLabs). It is well-known that secondary radiation damage effects are responsible for non-linearity in the high-exposure data points. Therefore the XFMS data is often fitted using the first 3–4 points in the linear region of the dose–response plot^24^. The rate constant, *k* (sec^−1^), was used to measure the reactivity of a side-chain towards hydroxyl radical-induced modification^4^ (Supplementary Tables 3 and 4).

XFMS peptide identification and analysis have been automated and enhanced by adopting the Byos® (Protein Metrics Inc) integrated software platform. Byos encompasses the Byonic™ MS/MS search engine and the Byologic® peptide analysis software. The Byos oxidative footprinting workflow integrates the identification of peptide modifications with the quantification of residue-specific modifications. Modification Fine Control™ allows for the simultaneous search for a high number of modifications without causing a combinatorial explosion and associated lengthy search times. Byologic automatically extracts ion chromatograms and reports the quantification of modifications relative to the unmodified peptide based on the extracted ion chromatograms. A typical workflow starts with processing a high-exposure tandem MS (MS/MS) file in Byos for an MS/MS search against FASTA sequences and the localization of modification sites. The peptide-level analysis and validation of assignments are carried out in Byologic and lead to the creation of in-silico peptides in the form of a CSV file using the MS/MS data. The in-silico peptides CSV is subsequently applied to full scan (MS1) data covering a series of exposure times, and the resulting quantified peptide modifications provide the basis for the residue-specific and peptide-level dose–response. The automated workflow outlined here has resulted in significant time savings in the data analysis step of XFMS.

### Reporting summary

Further information on research design is available in the Nature Research Reporting Summary linked to this article.

## Supplementary information

Supplemental Material

Description of Additional Supplementary Files

Supplementary Data 1

Supplementary Data 2

Reporting Summary

## Data Availability

The data generated or analyzed during this study are available in the Supplementary Tables and Supplementary Data files. Any additional data files are available from the corresponding authors on reasonable request.
