# Supplementary material for: An automated liquid jet for fluorescence dosimetry and microsecond radiolytic labeling of proteins
Source: Commun Biol. 2022 Aug 25;5:866. doi: 10.1038/s42003-022-03775-1 (PMC9411504; doi:10.1038/s42003-022-03775-1)
Supplement: Supplementary file 1 — Supplemental Material [file 42003_2022_3775_MOESM1_ESM.pdf]

An automated liquid jet for fluorescence dosimetry and microsecond radiolytic labeling of proteins

Mathew Rosi[1], Brandon Russell[1], Line G. Kristensen[2], Erik R. Farquhar[3], Rohit Jain[3], Donald Abel[3], Michael Sullivan[3], Shawn Costello[4], Maria Agustina Dominguez-Martin[5,6], Yan Chen[7], Susan Marqusee[4], Christopher J, Petzold[7], Cheryl A. Kerfeld[5,6], Daniel P. DePonte[8], Farid Farahmand[1], Sayan Gupta[2], Corie Y. Ralston[9]

[1]Sonoma State University, Rohnert Park, California 94928, United States
[2]Molecular Biophysics and Integrated Bioimaging Division, Lawrence Berkeley National Laboratory, Berkeley, California 94720, United States
[3]Center for Synchrotron Biosciences, School of Medicine, Case Western Reserve University, Cleveland, Ohio, 44106 United States
[4]Department of Molecular and Cell Biology, University of California at Berkeley, Berkeley California 94720, United States
[5]MSU-DOE Plant Research Laboratory and Department of Biochemistry and Molecular Biology, Michigan State University, East Lansing, Michigan 48824, United States
[6]Environmental Genomics and Systems Biology Division, Lawrence Berkeley National Laboratory, Berkeley, CA 94720, United States
[7]Biological Systems and Engineering Division, Lawrence Berkeley National Laboratory, Berkeley, California 94720, USA
[8]Stanford Linear Accelerator Center, Menlo Park, CA 94025, United States
[9]Molecular Foundry Division, Lawrence Berkeley National Laboratory, Berkeley, California 94720, United States

<u>Contents of Supplementary Information</u>

Supplementary Note 1 – contains Supplementary Figures S1 through S9

Supplementary Note 2 - contains Supplementary Figures S10 through S16

Supplementary Figure S17

Supplementary Figure S18

Supplementary Figure S19

Supplementary Figure S20

Supplementary Figure S21

Supplementary Table 1

Supplementary Table 2

Supplementary Table 3

Supplementary Table 4

Supplementary References

Supplementary Note 1. Automated system design

A user interface was created to communicate with the Fluorescence Imaging Module (FIM) that allows the user to control the experimental parameters and visualize the output data in real time. The system is programmed with LabVIEW, which is a graphical programming-based system engineering software that facilitates interaction and communication with various components, through many different communication protocols, in most cases through National Instruments (NI) Virtual Instrument Software Architecture (VISA). LabVIEW implements the use of graphical icons consisting of terminals, sub–Virtual Instruments (sub-VIs), functions, and structures that are interconnected by virtual traces.

Supplementary Note 1.1. Interface

The interface was designed to run on the footprinting beamlines at the Advanced Light Source, Lawrence Berkeley National Laboratory. To establish proof of concept and to facilitate realistic design specifications, the order of experimental operation for the automated instrumentation shown in Supplementary Figure 1 is performed as follows: a blue LED (1) is used to illuminate a water solution mixed with Alexa Fluor 488 dye that is expelled from the capillary tube (2) by the syringe pump (3). The light from the LED is reflected at 90 degrees and passes through the direction of the jet flow, illuminating the fluorescence in the solution. The fluorescence is reflected at the prism (4) mounted to the PMT, sending 90% of the light to the camera (5) and 10% to the PMT (6). The PMT voltage is read by the DAQ (7) and sent to the user interface. After each sample is processed the rotation stage (8) moves to the next container. By using different concentrations of the dye [0.5 – 5 µM], a dose response curve can be simulated that represents variable amounts of X-ray exposure.

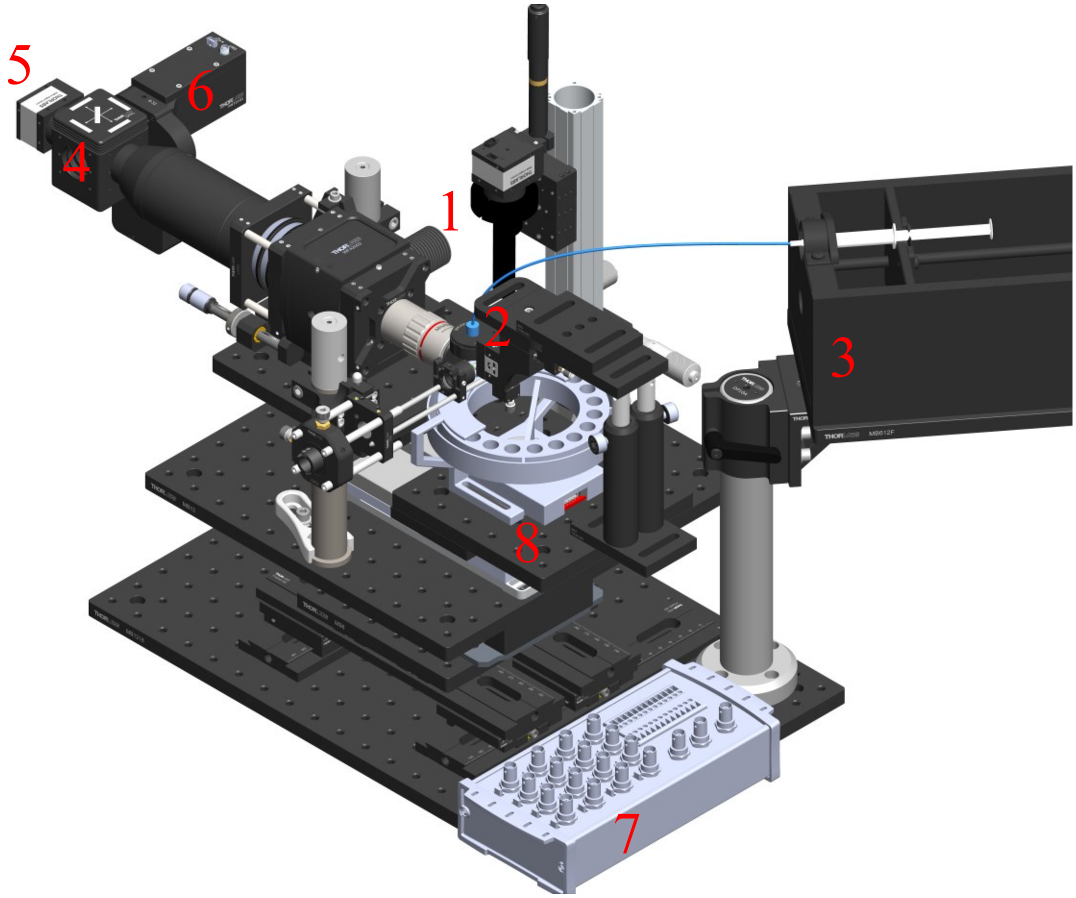

Supplementary Figure S1: Instrument design showing the critical components of the system. 1: LED, 2: Capillary, 3: Syringe Pump, 4: Prism, 5: PMT, 6: Camera, 7: DAQ, 8: Rotation Stage.

The control side of the interface (Supplementary Figure 2) has 5 user inputs that are used to determine the command that is sent to the syringe pump, in terms of volumetric flow rate ($\mathbf{Q}$). The experimental inputs are syringe diameter ($\boldsymbol{D_S}$), jet diameter ($\boldsymbol{D_J}$), X-ray exposure length ($\boldsymbol{L_E}$), X-ray exposure time ($\boldsymbol{t_E}$), and target volume ($\boldsymbol{V_T}$). X-ray exposure time is the critical experimental component that allows the user to determine the dose response curve of the buffer solution. X-ray exposure length refers to the physical vertical dimension of the beam and is a known and generally fixed dimension. Focusing mirrors can also be used to focus the beam to a specific energy density and size.

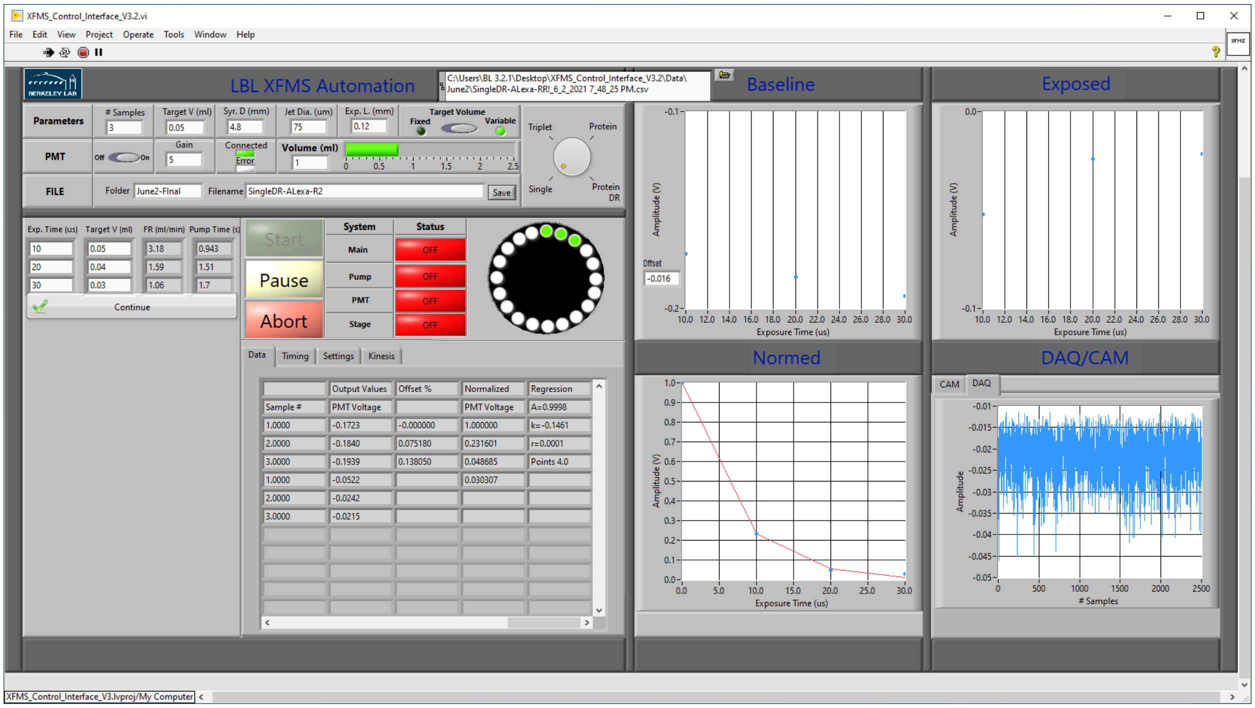

Supplementary Figure S2: GUI Experimental Control Panel. Configuration includes user inputs for number of samples, target volume, syringe diameter, jet diameter and exposer length. Exposure time is the experimental control user input and is entered for each sample with a maximum of 20 samples.

Since the command that the pump interprets is in terms of volumetric flow rate (ml/min) and the experimental input for each sample is in terms of exposure time (µs), a conversion function is integrated into the program. For visualization purposes a model is created to demonstrate the physical properties of the syringe (Supplementary Figure 3).

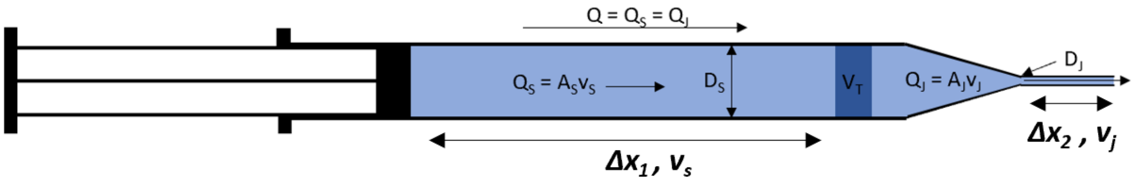

Supplementary Figure S3: Syringe Model. Showing pertinent dimensions to calculate volumetric flow rate (***Q***) at the exit point of the nozzle.

The exit velocity of the solution at the tip of the syringe is critical to the sample exposure time. The pump operates secondarily from the user input for the diameter of the syringe and it follows that the flow rate is derived from the exit velocity of the solution. The volumetric flow rate is

defined as the liquid volume that passes through a given cross-sectional area per unit time. The cross-sectional area is defined as the area of a two-dimensional shape contained in a three-dimensional object, or an infinitely small slice of the object. In the case where a change in area occurs, the Equation of Continuity is critical in determining the flow rate at a given point in the system. The equation of continuity follows the law of conservation of mass in fluid dynamics and is derived as follows (variable subscripts s and j refer to the syringe and jet, respectively):

Evaluating at short intervals of time (Δt), the fluid moves a linear distance of $\mathbf{\Delta x_1}$ at a velocity $\mathbf{v_s}$. The distance covered by the fluid in the barrel of the syringe is given by:

$$\mathbf{\Delta x_1 = v_s \Delta t}$$

The volume that flows through the uniform area in the barrel of the syringe is given by a function of area and distance in the equation:

$$\mathbf{V_s = A_s \Delta x_1 = A_s v_s \Delta t}$$

Since mass ($\boldsymbol{m}$) equals density ($\boldsymbol{\rho}$) times volume (V), the mass of the fluid in the barrel is given by the equation:

$$\mathbf{m_s = \rho_s A_s v_s \Delta t}$$

The mass flux is calculated and is defined as the mass of the fluid per unit time passing through a cross sectional area. The mass flux in the uniform barrel area of the syringe is given by:

$$\mathbf{\Delta m_s / \Delta t = \rho_s A_s v_s} \qquad \textbf{(2)}$$

The mass flux at the tip of the syringe that represents the capillary tube where the fluid is ejected, with velocity $\mathbf{v_j}$, is given as:

$$\mathbf{\Delta m_j / \Delta t = \rho_j A_j v_j} \qquad \textbf{(3)}$$

The density of the fluid remains the same at both cross-sectional areas at a constant velocity. Therefore, the barrel mass flux **(2)** is equal to the jet mass flux **(3):**

$$\rho_s A_s v_s = \rho_j A_j v_j$$

The fluid can be assumed as incompressible since any pressure changes associated in the flow are negligible. This means that the fluid density in the barrel is the same in the jet:

$$A_s v_s = A_j v_j \qquad (4)$$

The volumetric flow rate (***Q = ml/min***) is in terms of volume (***V***) per unit time (***t***), and is extrapolated from the generalized form of the mass flux equation:

$$Q = Av = m^2 m s^{-1} = m^3 s^{-1}$$

$$m^3 s^{-1} \times 60\ s\ min^{-1} \times 10^6\ ml\ m^{-3} = ml\ min^{-1}$$

The user input of exposure time (t_E), in microseconds, is divided by the exposure length (L_E), in millimeters to determine the appropriate jet velocity in meters per second that sets the appropriate flow rate:

$$v_j = 10^{-3} L_E 10^6 t_E^{-1} = ms^{-1}$$

$$Q = A_J v_J$$

Supplementary Note 1.2. Components

The required experimental system components include: National Instruments USB-6216 Data Acquisition device, Thorlabs PMT2101 Photomultiplier Tube (PMT), Thorlabs SH1 Optical Beam Shutter and KSC101 Compact Shutter Controller, Thorlabs ELL18 Rotation Stage with Resonant Piezoelectric Motors, Harvard Apparatus PHD ULTRA 4400 Syringe Pump, and Basler ace acA640-120gc Color GigE Camera.

Supplementary Note 1.2.1. Syringe pump

The Harvard Apparatus PHD ULTRA 4400 is a high accuracy, bi-directional syringe pump with infuse and withdrawal capabilities. The pump is driven by a 1.8-degree stepper motor that generates 200 pounds of force. Communication is achieved over the USB protocol, at a 9600 baud

rate, through NI VISA. Typically, VISA mandates that a COM port is selected by the user; however, to simplify the program, an automatic device recognition sub-VI is created that queries the software version of the instrument and checks against a pre-compiled list to assign a COM port that will be used in relevant functions. NI provides configuration sub-VI's to configure and operate the pump that converts user inputs to string commands sent through the interface to set pump direction, flow rate, and syringe diameter.

Supplementary Note 1.2.2. Rotation stage

The sample tubes are situated in a custom designed tray mounted to the Thorlabs ELL18 Rotation Stage driven by resonant piezoelectric motors. The stage is positioned under the fixed capillary tube where the syringe pump expels solution. The stage specification for rotational positioning is 43.0 μrad and is sufficient resolution to produce repeatable dispensing in the 1 cm target area of the tube opening. At the initialization stage of the main program, the stage is configured for USB protocol using NI VISA. During the initialization period, a sub-VI is used to set the stage to a home position of zero degrees and is physically achieved using an infrared reflecting optical sensor for positioning within 0.5 – 1 mm and a magnetic sensor to achieve 1-micron resolution. A provided USB driver allows string commands to be sent by LabVIEW to move the stage after each sample is processed.

Supplementary Note 1.2.3. Photomultiplier tube

The fluorescence from the Alexa Fluor 488 nm dye, contained in the sample solution, emits fluorescent light that is read by the Thorlabs PMT2101 Photomultiplier Tube (PMT). The 488 nm wavelength is well-suited for the PMT, as it falls within the optimal photocathode radiant sensitivity range (Supplementary Figure 4). A photocathode collects the light as an input to the integrated gallium arsenide phosphide (GaAsP) transimpedance amplifier that is designed to

amplify signals from 0-80 MHz with a min/max input voltage of ±1.5 V. The PMT is controlled by utilizing a pre-configured Dynamic Link Library (DLL) provided by Thorlabs. The DLL is compiled in C++ and a Call Library Function Node is implemented as a sub-VI to send function calls to the PMT.

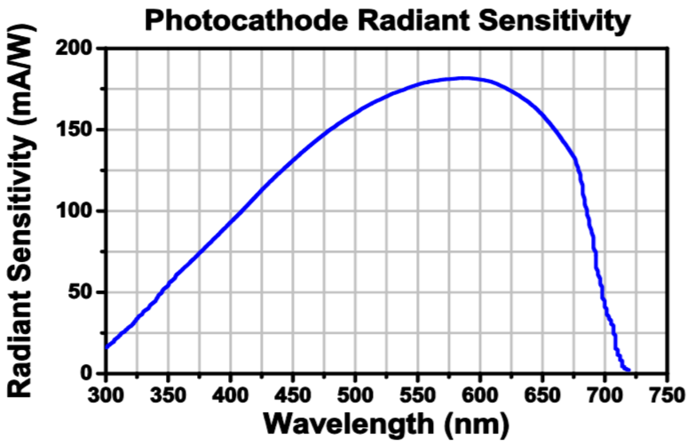

Supplementary Figure S4: Photocathode radiant sensitivity. Graph showing that the PMT is well suited to detect emission wavelengths of 488 nm. Source: Thorlabs PMT2100 Series Photomultiplier Tubes User Guide.

Supplementary Section 1.2.4. Optical beam shutter

The Thorlabs SH1 Optical Beam Shutter is a rotary electro-mechanical actuator that provides millisecond shutter operation. The shutter is placed directly in line with the PMT and is actuated at the precise moment when solution is dispensed from the jet to ensure accurate readings while minimizing delay. The shutter is operated with a Thorlabs KSC101 K-Cube Solenoid Controller and Thorlabs Kinesis .NET Framework. The solenoid controller has a trigger input that receives a digital signal from the DAQ. When the signal is received, the shutter opens to allow the PMT to measure the light intensity. This combination allows for activation of the shutter with minimal signal transmission delay. Supplementary Figure 5 shows the timing of the shutter, where TI represents the response time from a voltage signal to the start of the shutter actuation (10 ms). TO represents the time taken for the shutter to reach 80% open aperture (10 ms). TD/R is the time

required for the shutter to begin the close sequence after the voltage source is removed (20 ms). TC represents the

response time for the aperture to reach a 20% closed status (10 ms). MSOP is the minimum shutter opening time (40 ms). MOP is the minimum opening pulse (40 ms).

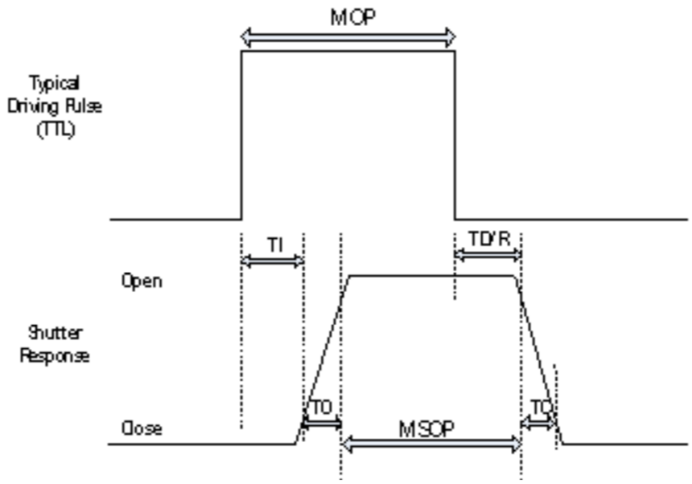

Supplementary Figure S5: Optical Beam Shutter Timing Diagram. Source: Thorlabs SH1 and SH1/M Ø1" Beam Shutter User Guide.

<u>Supplementary Note 1.2.5. Data acquisition device</u>

The voltage from the PMT is read by an analog input on a National Instruments USB-6216 Data Acquisition (DAQ) device. The DAQ has a maximum sampling rate of 400 kSs^{-1}, 16-bit DAC, and 50 ns timing resolution. A digital output is used to trigger the optical beam shutter and to simulate data acquisition timing to ensure accurate data is collected (for development purposes only). The DAQ communicates with LabVIEW over USB and a configuration-based tool called DAQ Assistant from the NI-DAQmx data acquisition driver package is used to establish the I/O channels.

<u>Supplementary Note 1.2.6. GigE camera</u>

A Basler ace acA4024-8gc Color GigE Camera is used to monitor the inline fluorescence emitted by the liquid jet delivery system (Supplementary Figure 6). Ensuring that the jet is in focus with

the camera will allow the PMT to capture accurate results, since both have the same working distance. The camera is GigE Vision Standard compliant and interfaces with LabVIEW via ethernet (RJ45) and NI Vision Acquisition Software.

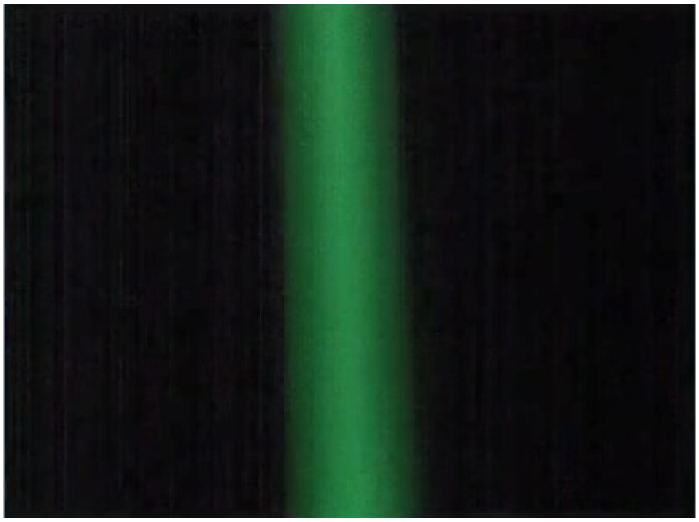

Supplementary Figure S6: GigE Camera View of Solution. Inline view of fluorescence emitted from the solution expelled through a 50 µm ID capillary tube.

Supplementary Note 1.3. Timing

Timing is critical for the operation of the interface, as well as the experimental data acquisition. The pump runs for a given time depending on the flow rate, which is calculated for each sample iteration and ensures that data is captured precisely for each iteration. The order of operation is defined in a sequence structure based completely on variable sample-dependent timing. During testing it was found that a small bubble can form at the tip of the capillary tube that registers a voltage that exceeds the limits of the PMT and causes it to enter an error state, yielding unreliable results. This is a significant problem because it takes two iterations for the PMT to reset and valuable data and sample solution are lost. To mitigate this risk, the PMT is programmatically set to turn on one quarter of the way through the pump cycle and turn off at three quarters, for a maximum PMT operation time of one-half the pump time. The drawback to this solution is that it

substantially increases the minimum pump time proportionately with the sample volume needed for each iteration.

In each iteration of the main sequence, the amplifier to the PMT is turned on and off corresponding to the data acquisition protocol. It was found that response time after the amplifier was turned on and until a stabilized reading could be achieved was ~ 225 ms (Supplementary Figure 7). A similar anomaly occurred when the amplifier was turned off, although stabilization time was ~ 25 ms. The minimum samples needed to acquire an accurate measurement from the PMT was set at 2500 samples, combined with the sampling rate of the DAQ (400kS/s) designated a minimum sampling time of 6.25 ms plus 40 ms of inherent software delay for each cycle. These time constraints established that the minimum achievable pump time would be set at 600 ms (Supplementary Figure 8), including additional buffer delay, resulting in a minimum sample volume of 50 μL.

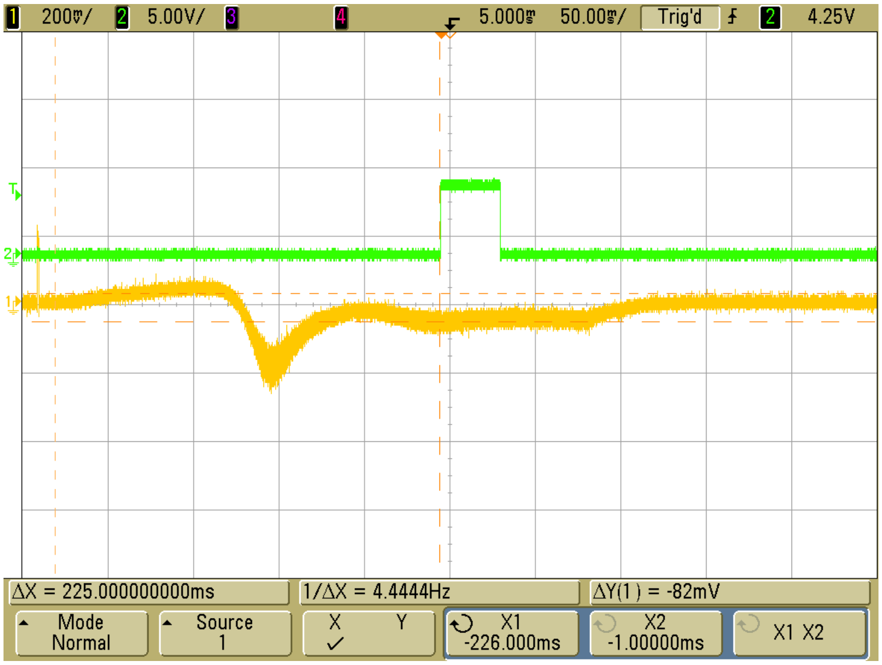

Supplementary Figure S7: PMT Oscilloscope Reading. PMT response (yellow), with simulated data acquisition timing (green). The PMT amplifier turns on at the spike occurring at ~ t = 10 ms.

Minimizing the volume sample solution was a key design specification of the system and with the minimum pump time of 600 ms, the minimum sample volume was determined to be 50 µL. A determination was made that smaller sample sizes could be achievable with the use of an inline optical beam shutter. This allowed for the possibility to keep the PMT amplifier on, thus eliminating the required PMT stabilization time. The shutter activation/deactivation time of 50 ms, combined with the 50 ms data acquisition time resulted in a PMT activation time of 150 ms and a minimum overall pump time of 300 ms, reducing the minimum sample volume to 25 µL (Supplementary Figure 9).

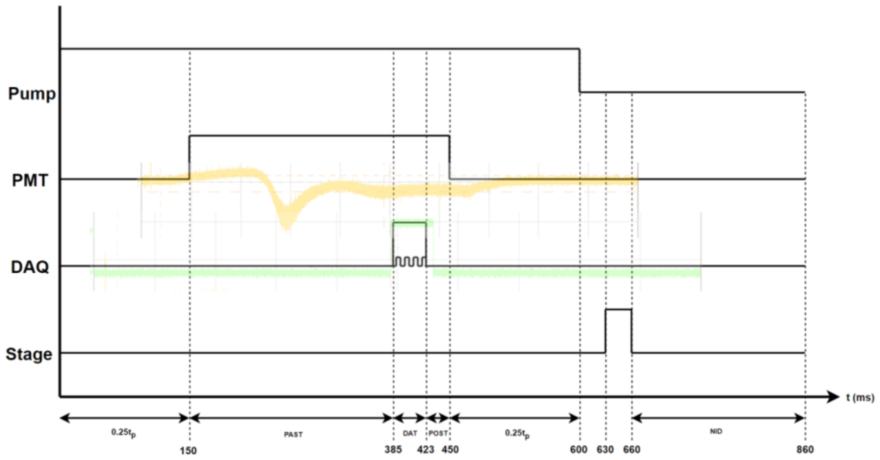

Supplementary Figure S8: Instrumentation Timing Diagram (No Shutter). System timing diagram with PMT and DAQ signals overlaid for visualization purposes, showing the minimum overall pump time of 600 ms.

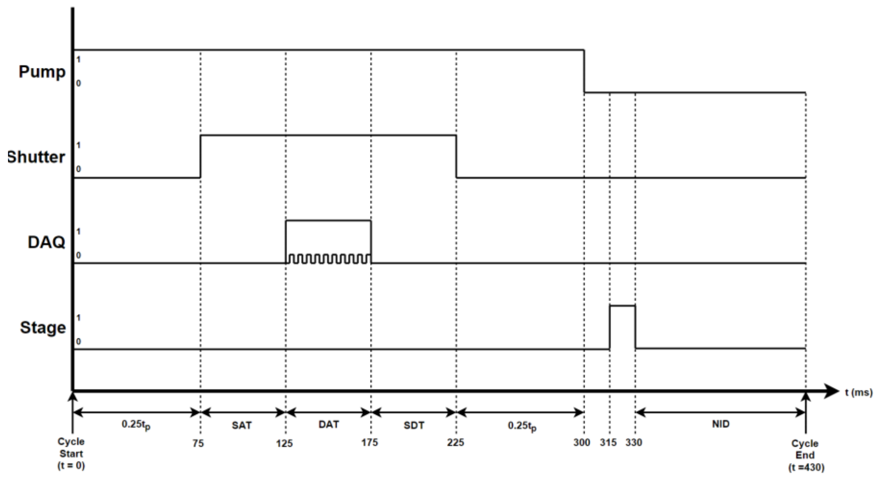

Supplementary Figure S9: Instrumentation Timing Diagram w/ Shutter. For one cycle, shown with minimum pump time (t_p) of 300 ms. Each timing variable can be adjusted manually to facilitate procedural experimentation. Timing variables are listed as SAT: Shutter Activation Time (50 ms), DAT: Data Acquisition Time (50 ms), SDT: Shutter Deactivation Time (50 ms), NID: Next Iteration Delay (100 ms).

Supplementary Note 1.4. Automated Alexa Dose Response Analysis

We have two modes of dose-response analysis: Single DR mode and Protein DR mode. In the Single DR mode, PMT data are collected twice for a set of identical progressive increases in the jet velocity, one with keeping the beam off and the other with the beam on. The PMT data with beam off is used as baseline data for respective jet velocities (exposure times). The jet is usually focused at its highest velocity using the FIM, which has a narrow depth of field. The changes in the jet velocity affect the focusing, which changes the PMT reading. The PMT reading increases by 0 to 25 %, depending on the extent to which the jet is shifted for the FIM's optimum depth of focus. To compensate for the effect of this increase in the PMT reading, we determine the % change values from the beam off PMT reading for each jet speed with respect to the fully focused jet (the highest speed). These % change values obtained from the first set are used as correction factors for the PMT values for individual jet speed in the second set (beam on). The PMT dark (LED off) value is then subtracted from the corrected PMT (beam on) values to remove the effect of background scattered lights. Finally, the PMT data of the exposed sample is normalized with

respect to the "zero" exposure to obtained fraction unmodified vs. exposure time. The data is then analyzed with single exponential nonlinear regression.

Supplementary Note 2. Automated beam alignment

The automated instrumentation system was designed to be used for XFMS experiments primarily at U.S. Department of Energy (DOE) funded, synchrotron-based, national research facilities. This system has been tested thus far at Beamline 17-BM XFP, at the National Synchrotron Light Source II (NSLS-II), Brookhaven National Laboratory (BNL) and Beamlines 3.2.1 / 5.3.1 at the Advanced Light Source (ALS), Lawrence Berkeley National Laboratory (LBNL). Synchrotrons have multiple beamlines, each at different energy densities. Moreover, each synchrotron facility has different technical specifications that govern the X-ray beam properties. It is important in XFMS to quantify the irradiated dose delivered to the samples to establish a relationship between site modifications and dose energy exposure. The relationship is expressed in flux (photons/sec/mrad^2) as a function of beam energy (keV). Since a beamline energy is known at a given facility, this can be determined easily. However, due to the distribution of photons in the beam, it is important to ensure that liquid jet sample delivery is centered at the highest concentration beam photon density. Development of the methodology to facilitate beam/jet alignment requires consideration of the characteristics for each beam and as such, a variable independent method is developed.

Supplementary Note 2.1. Beam alignment module (BAM)

To facilitate an automated approach with minimal intervention, a 0.75x objective with doubling tube and attached camera is mounted to a 12 mm travel vertical linear stage actuator. An ND YAG is mounted on an apparatus situated ~ 3 mm from the jet. A fluorescent pattern manifests when the X-ray contacts the ND YAG. The light from the jet and the beam pattern is reflected at 90 degrees to the vertical objective lens (Supplementary Figure 10). This is a necessary configuration due to

risk of equipment damage from the high energy density of the beam. Since highly focused images of the beam and jet cannot be viewed simultaneously, the vertical stage is used to alternate focus on both points of interest. A Basler ace acA1300-30gm Color GigE Camera is mounted to the lens and features a pixel height and width of 1.3 µm. A Python wrapper for the Basler Pylon Camera Software Suite is integrated into the software with a limitation of 1500 bytes per frame maximum transmission rate and image size of 1024 X 1280 (H X W). The BAM components are integrated and situated onto an independent linear stage actuator that allows for precise beam alignment based on the results from the image processing algorithm.

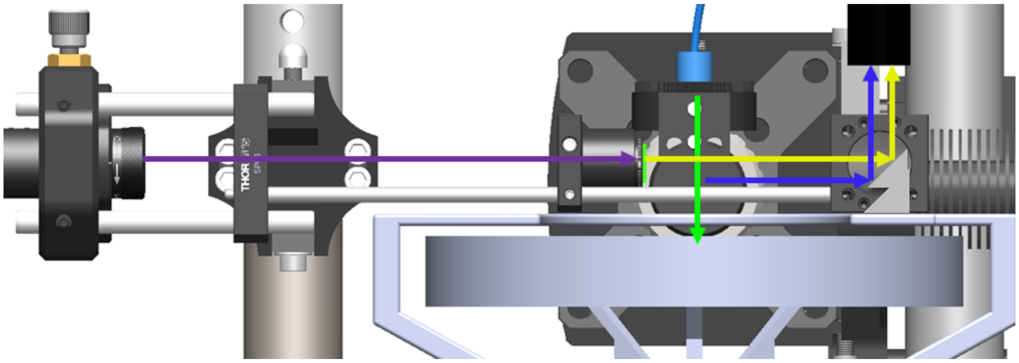

Supplementary Figure S10: View of alignment system, orthogonal to the X-ray beam plane. The system is configured such that the beam exposes the sample solution ~ 2 cm above the linear view of the PMT, which is the center of the objective lens below the jet. The blue led reflected through the objective lens causes the jet to appear blue in the camera view.

Supplementary Note 2.1 Alignment methods

Digital image processing is implemented using a variety of techniques suited to detect the relevant features of the X-ray and the liquid jet. The techniques are performed using Python and OpenCV. The OpenCV library for Python acts as a wrapper for computationally intensive C/C++ codes and are implemented as Python functions. OpenCV Python functions are combined with functions from the Numpy scientific computing package, designed for multidimensional arrays, and allows for efficient image processing.

Supplementary Note 2.2 Beam center detection

The unprocessed beam image (Supplementary Figure 11) is captured and converted to an array. The first step in the image processing algorithm is to reduce any noise in the image by applying a Gaussian smoothing filter on the converted, single channel grayscale image array. A Gaussian filter is a special weighted averaging filter and is employed in several image processing tasks such as image blurring, image segmentation, and edge detection [1].

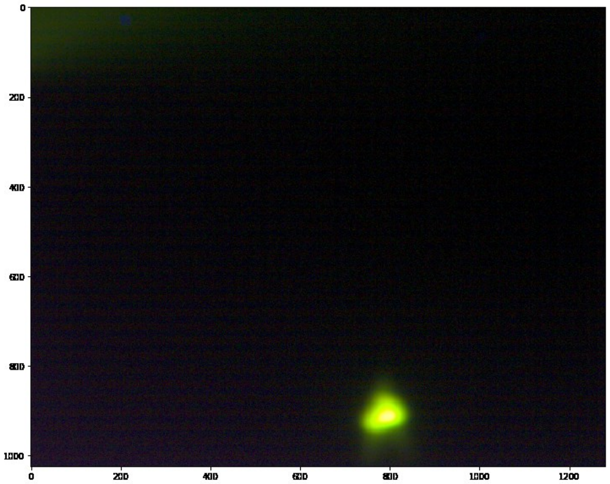

Supplementary Figure S11: Fluorescent Beam Image. Unprocessed image of beam pattern in contact with ND YAG.

The next, and most critical step is to pass the image array through a thresholding function that sets pixels above a given value as 255 and below the threshold value as zero. This is challenging, since environmental and/or experimental conditions are dynamic, thus creating the potential for different image values from one experiment to the next. This means that choosing an arbitrary threshold value based on a set of tests conducted during development is not optimal. Otsu's method, which is widely used automated image thresholding technique, is chosen to optimize this procedure. Image binarization is achieved through Otsu's method by finding a threshold value (***T***), between two distinct regions of the image, that reduces the weighted variance of the image object given by the relationship:

$$\sigma_w^2(T) = q_1(T)\sigma_1^2(T)q_2\sigma_2^2(T)$$

Where $\boldsymbol{q_1}$ and $\boldsymbol{q_2}$ represent the distinct color regions and are defined as:

$$q_1(T) = \sum_{i=1}^{T} P(i)$$

$$q_2(T) = \sum_{i=T+1}^{I} P(i)$$

Where the weighted means are calculated as:

$$\mu_1(T) = \sum_{i=1}^{T} iP(i)\,[q_1(T)]^{-1}$$

$$\mu_2(T) = \sum_{i=T+1}^{I} iP(i)\,[q_2(T)]^{-1}$$

And the variance is given by:

$$\sigma_1^2(T) = \sum_{i=1}^{T} [i - \mu_1(T)]^2 P(i)\,[q_1(T)]^{-1}$$

$$\sigma_2^2(T) = \sum_{i=T+1}^{I} [i - \mu_2(T)]^2 P(i)\,[q_2(T)]^{-1}$$

Where the threshold value is clearly defined between the two variance values and results in clearly defined edges of the beam as shown in Supplementary Figure 12.

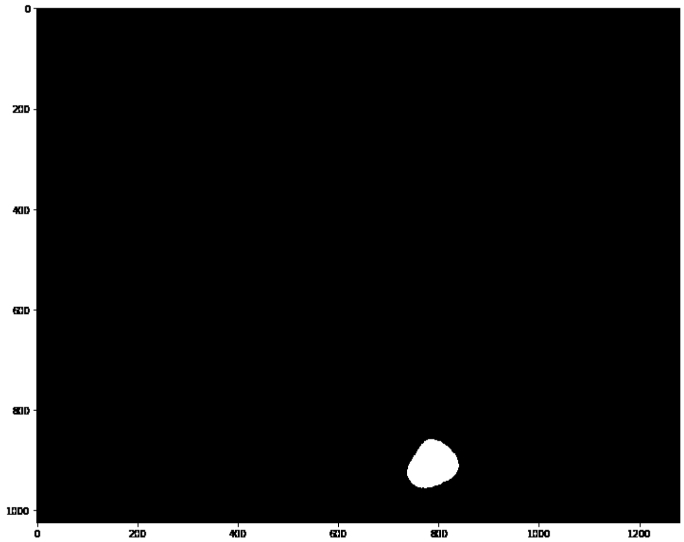

Supplementary Figure S12: Binarized Beam Image. Threshold binarization is achieved through Otsu's method and creates clearly defined binarized boundaries of the beam in the image.

The resulting image consists only of tightly grouped pixels that share the same values where image moments can be extracted from the object. Analog images can be modeled using a Cartesian distribution function [$\boldsymbol{f}$ **(*x, y*)**] that can be implemented to determine the **(*p* + *q*)**$^{\mathbf{th}}$ order image moments with the basis function ($\boldsymbol{\psi_{pq}}$) over the image plane (***P***), represented in the equation:

$$M_{pq} = \iint_P \psi_{pq}(x,y) f(x,y) dxdy; \quad p,q = 0,1,2,\ldots,\infty$$

This equation results in a weighted average over the image plane, where the basis function is designed, such that it is representative of invariant features of the image and the properties are passed onto moments [2]. In digital signal processing the image is represented with binary pixel intensities, [***I(x, y)***] in a discrete function where the two-dimensional **(*p* + *q*)**$^{\mathbf{th}}$ order digital Cartesian moments are defined as [3, 4]:

$$M_{pq} = \sum_{x=1}^{K} \sum_{y=1}^{L} x^p y^q I(x,y)$$

Over the image dimensions ($\boldsymbol{K}, \boldsymbol{L}$) and where the basis function is now represented as $\boldsymbol{x^p y^q}$. The first order moments are used to find the x and y coordinates of the centroid of the pixel cluster (object):

$$\bar{x} = \frac{M_{10}}{M_{00}} \;,\; \bar{y} = \frac{M_{01}}{M_{00}}$$

This results in a clearly defined center point of the beam as shown in Supplementary Figure 13. The x and y pixel coordinates of this location are saved as a reference to the beam center.

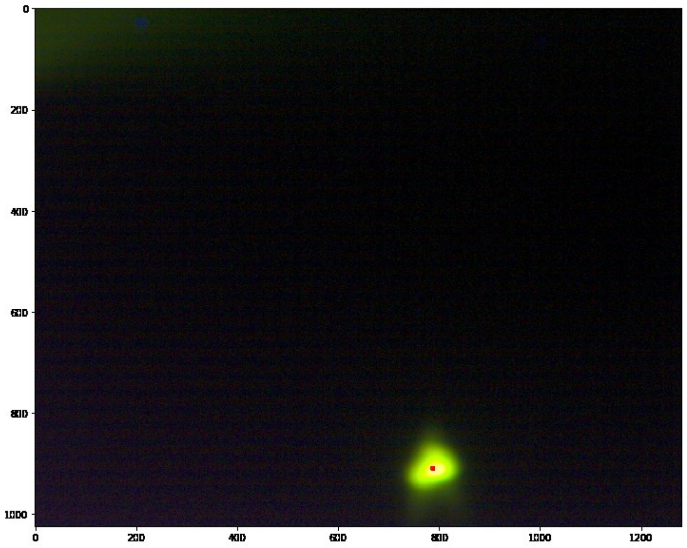

Supplementary Figure S13: Beam Center Point. Result of beam center detection, with center point plotted for visualization.

<u>Supplementary Note 2.3. Liquid jet center detection</u>

Once the center of the beam is found, it is considered fixed, and the linear stage actuator positions the objective precisely at the distance needed to observe the reflection of jet. The liquid jet is monitored throughout the duration of the experiment to ensure that each sample is receiving the expected irradiated dose. Since the beam is also constantly in the field of view (although unfocused), the green and red channels are removed from the image to prevent disruption to the jet center detection algorithm. Like the beam center detection algorithm, the image is converted to grayscale and a thresholding operation is performed (Supplementary Figure 14).

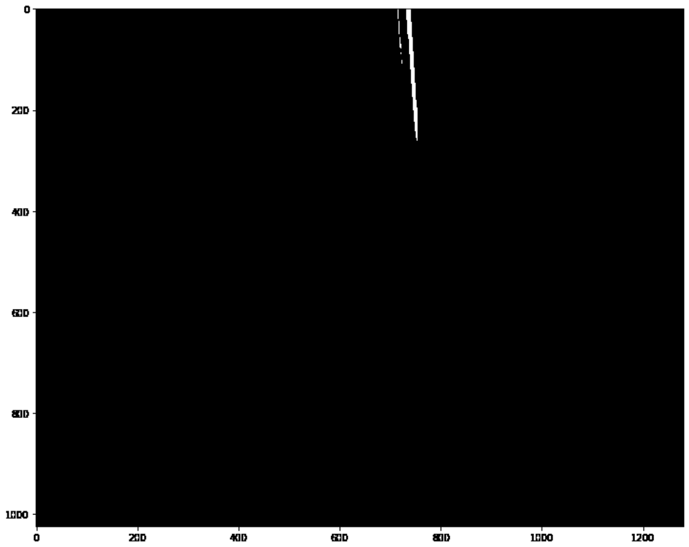

Supplementary Figure S14: Jet Image Binarization. Due to the relatively small field of view, only a portion of the jet is visible to the camera, which is a result of the reflected blue LED light. Note that the images are inverted.

To detect the center of the jet, the extreme edges of the image must be detected so that coordinate operations can be performed on the threshold image. This is achieved using the Canny edge detection algorithm, which is an accurate method of detecting edges in an image. The process involves a multi-stage algorithm [5, 6] that includes noise reduction, finding the intensity gradient, suppressing non-maximum points, and hysteresis thresholding. Edge detection calculations rely on derivatives, thus every pixel in the frame is taken into consideration and calculations are highly sensitive to noise. The noise in the image is reduced by sliding a 5 X 5 Gaussian kernel ($\boldsymbol{K_G}$) over the image, where the kernel is divided by the sum of the values in the distribution:

$$K_G = \frac{1}{159}\begin{bmatrix} 2 & 4 & 5 & 4 & 2 \\ 4 & 9 & 12 & 9 & 4 \\ 5 & 12 & 15 & 12 & 5 \\ 4 & 9 & 12 & 9 & 4 \\ 2 & 4 & 5 & 4 & 2 \end{bmatrix}$$

The intensity gradient ($\boldsymbol{G_x, G_y}$) calculation detects the intensity and direction of image edges, which correspond to the localized change in a pixel's intensity, by convolution of the image array ($\boldsymbol{I}$) with 3 X 3 Sobel kernels:

$$G_x = I * \begin{bmatrix} -1 & 0 & 1 \\ -2 & 0 & 2 \\ -1 & 0 & 1 \end{bmatrix}, \quad G_y = I * \begin{bmatrix} -1 & -2 & -1 \\ 0 & 0 & 0 \\ 1 & 2 & 1 \end{bmatrix}$$

Where the magnitude (***G***) and phase (***θ***) of the gradient are given by:

$$|G| = \sqrt{G_x^2 + G_y^2}, \quad \theta = \tan^{-1}\left(\frac{G_y}{G_x}\right)$$

Non-maximum suppression is performed to thin out the remaining filtered edges so that they are easily identifiable. The process involves processing all points from the gradient intensity matrix and finds pixels with local maxima in the direction of the edges. With these points identified, the process of hysteresis thresholding is performed, which involves setting faint pixels that are joined to bright pixels, as the same value as the bright pixel value that it is connected to. Due to the process of performing the Canny algorithm on an image with a performed threshold function, all bright pixels in the image result in the same value after processing. This process is needed to avoid any processing errors related to time, due to the high-speed trajectory of the jet solution.

While the Canny algorithm is useful for detecting edges, there are not any physical properties that can be derived from the calculation, i.e., edge coordinates. To facilitate this requirement, a technique known as Hough Transform is performed. This transformation can be used to detect any shape that can be represented in a mathematical coordinate system, even if the features are not clearly defined. Since vertical lines cannot be interpreted in the standard slope intercept for of the line equation, the transformation uses the parametric form:

$$\rho = x\cos\theta + y\sin\theta$$

$$y = -\frac{\cos\theta}{\sin\theta}x + \frac{\rho}{\sin\theta} \ \mid \ \theta \in [0{,}180], \ \ \rho \ \in \mathbf{R} \quad \textbf{(5)}$$

An empty 2-dimensional accumulator array is generated to hold values, where rows are denoted by $\boldsymbol{\rho}$ and columns denoted by $\boldsymbol{\theta}$**.** For an accuracy of one degree ,180 columns are needed, and the maximum number of rows is set to the diagonal length of the image. For each $\boldsymbol{x}$ and $\boldsymbol{y}$ coordinate

for the non-zero-pixel values in the image array, the parametric line equation is swept for all angles of $\boldsymbol{\theta}$ from 0-180 degrees. For every other non-zero-pixel found during each sweep, the accumulator array is upvoted in the corresponding row and column. At the end of the transformation the accumulator array is filtered by a program-defined minimum vote that returns all detected lines in the image. This process is computationally expensive, thus the OpenCV based Probabilistic Hough Transform function is used to maximize performance. The probabilistic version takes a random subset of the points, which is optimized by decreasing the threshold operation on the image. The result is a 4 x N dimension array represented by the end coordinates of the start and end points of each line that typically produces many lines. Due to the limited field of view and subsequent reduced visible portion of the jet, only a single line is detected from the brightest side in which the jet is closest to the LED. The center of the jet is calculated by converting half of the physical width of the jet to a pixel value and setting this as the center value. The center pixel value is plotted for visualization purposes (Supplementary Figure 15). The pixel difference between the center of the X-ray and the center of the jet is converted to physical distance using the pixel width of the camera sensor. The angle of the jet is used to calculate the offset from the two center points. In Supplementary Figure 16 the jet is perfectly centered, by calculating the distance of the two coordinates and sending the information to the linear stage actuator that the BAM is situated upon.

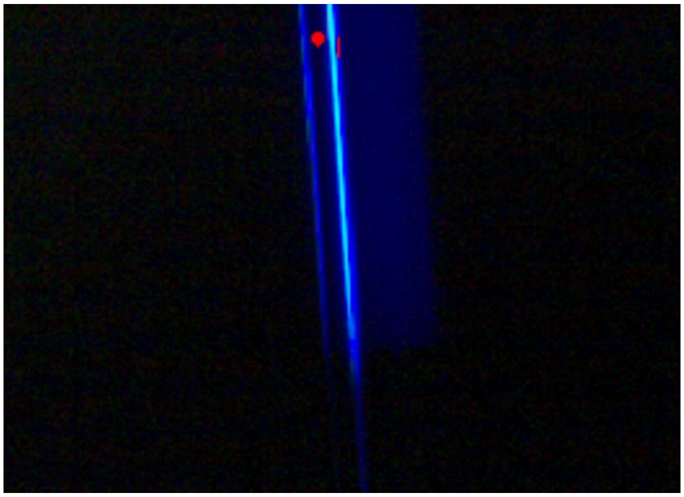

Supplementary Figure S15: Jet Center Point. Result of jet center detection method, with center point plotted for reference.

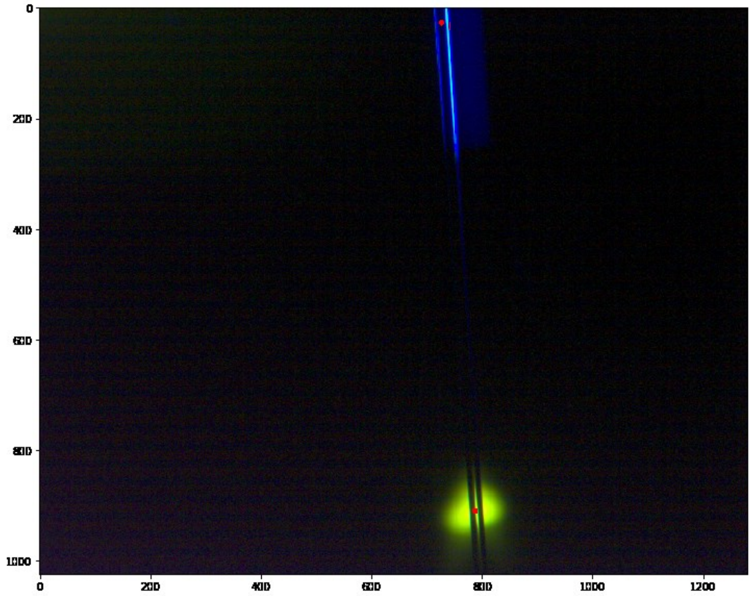

Supplementary Figure S16: Result of Alignment Method. All detection points are shown for reference.

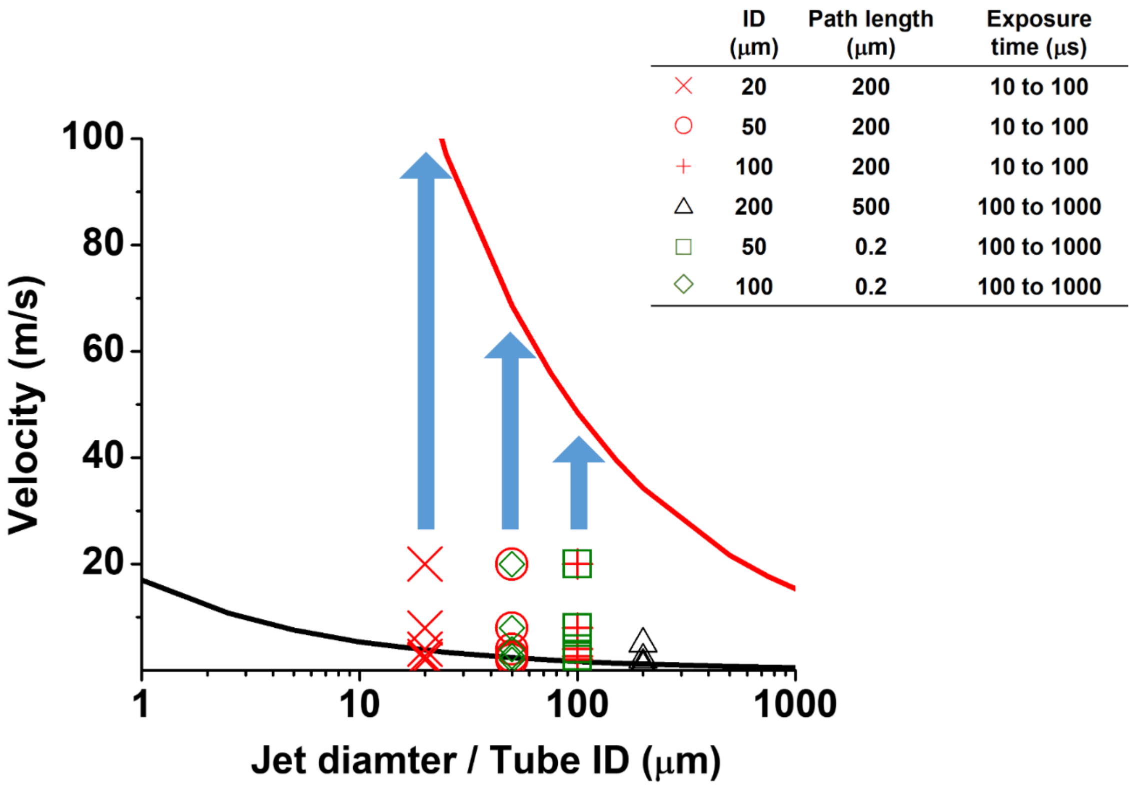

Supplementary Figure S17. This figure is reproduced from our previous publication [7] to show that the XFMS jetting regime is defined by flow speed, exposure time, and area of exposure and to show that a much wider choice of flow velocity is available with narrower jets. Jetting regime is shown within the lower (black) and upper (red) limit of flow velocity determined from the Weber number as a function of jet diameter as described previously. The individual points represent the optimum range of speeds for a given sample diameter and beam size for sample exposure. The colored set of points indicates the flow velocity that can be selected for the XFMS study at the unfocused beamline (green and black) and micro-focused beam (red) at ALS and NSLS-II. The blue arrow indicates a region of higher flow velocity that will provide shorter exposure, which will be useful to minimize sample damage as well as effectively use the higher flux density at a micro-focused beamline.

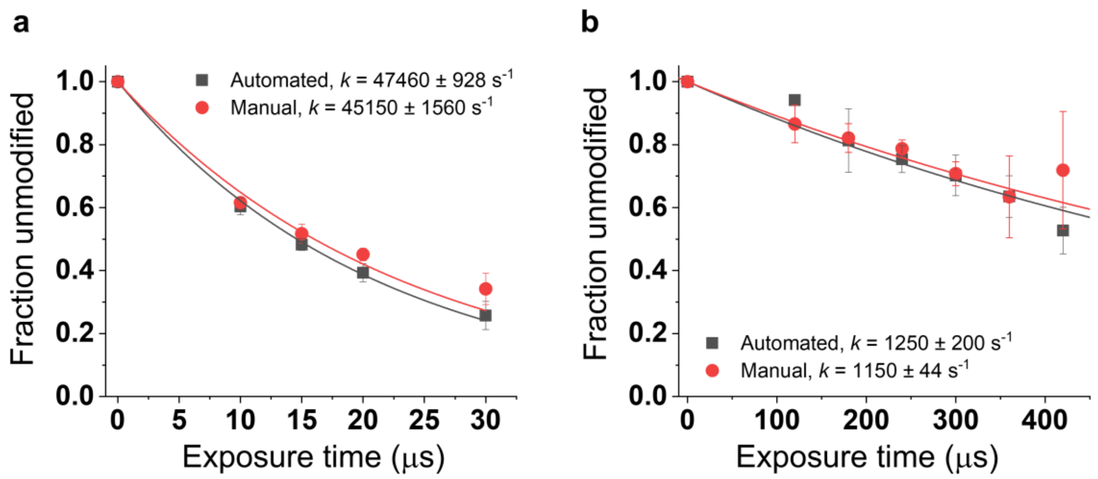

Supplementary Figure S18. Alexa dose-response plot obtained by automated and manual Alexa fluorescence measurements using a standalone fluorescence spectrometer for exposed samples at micro-focused high flux density beamline 17-BM at the NSLS-II (a) and unfocused low flux density beamline 3.2.1 at the ALS (b). Solid lines are single exponential fits using rate constants k (s^{-1}) as indicated in the figure.

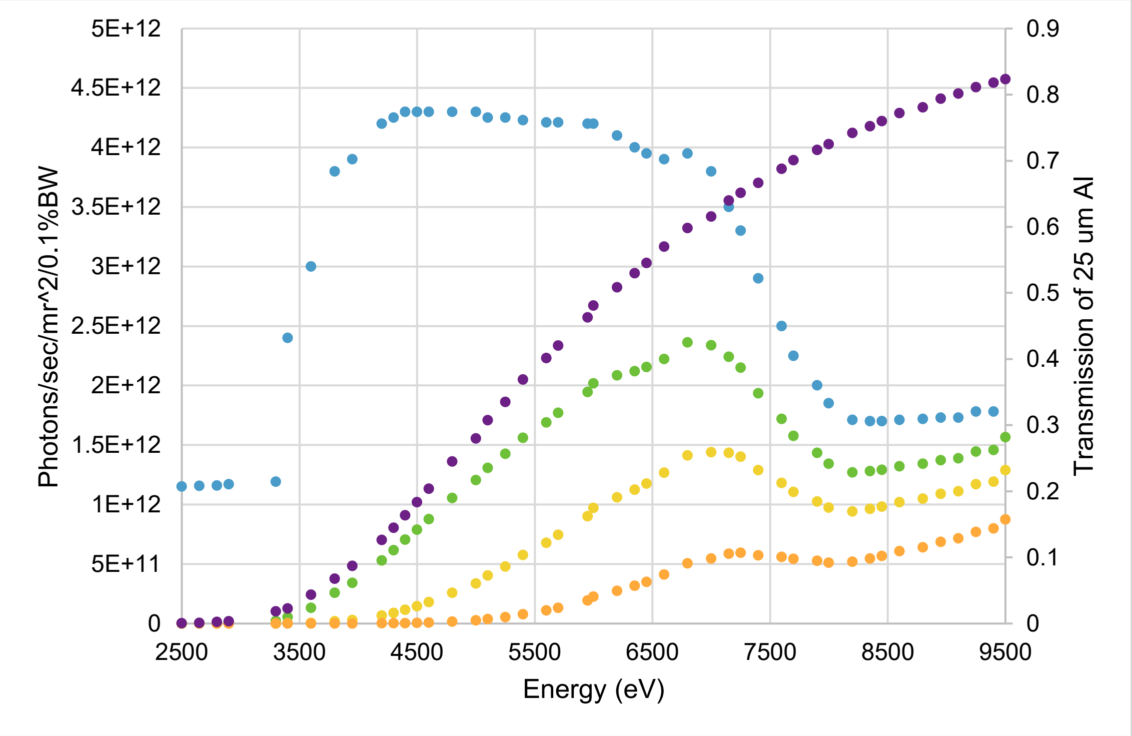

Supplementary Figure S19. Spectra for ALS beamline 5.3.1 with various Aluminum (Al) attenuation factors. Blue: Flux spectrum from internal LBNL report LSBL-624-531, "Beamline 5.3.1 Spectrally-Resolved Flux" by Ernie Glover, Phil Heimann, and Bob Schoenline, detailing measurement of beamline 5.3.1 flux using a calibrated silicon diode (International Radiation detectors AXUV-100 with 40 micron active layer thickness). Beamline 5.3.1 is located on a bend magnet source, with a vertically-deflecting platinum-coated 1:1 toroidal focusing mirror of length 900 mm, and a monochromator which is moved out of the beam path for footprinting experiments. The transmission plot for 25 micron thickness Al is shown in purple, as obtained from cxro.lbl.gov, with units on the secondary vertical axis on the right. The resulting transmitted flux for 25, 50, 100 and 200 micron thickness of Al are shown in green, yellow, orange, and red, respectively. Since Al attenuates less at the higher energies, the spectra shift up in wavelength with increasing Al attenuation. Using the transmitted flux curves shown here, and the geometry of the jet sample relative to the beam shown in Supplementary Figure S17, the absorbed energy in W (Joule/sec) for a 100 micron thick water sample was calculated as 2.9, 0.73, 0.33, 0.12, and 0.03 W for attenuations of 0, 25, 50, 100 and 200 microns, respectively.

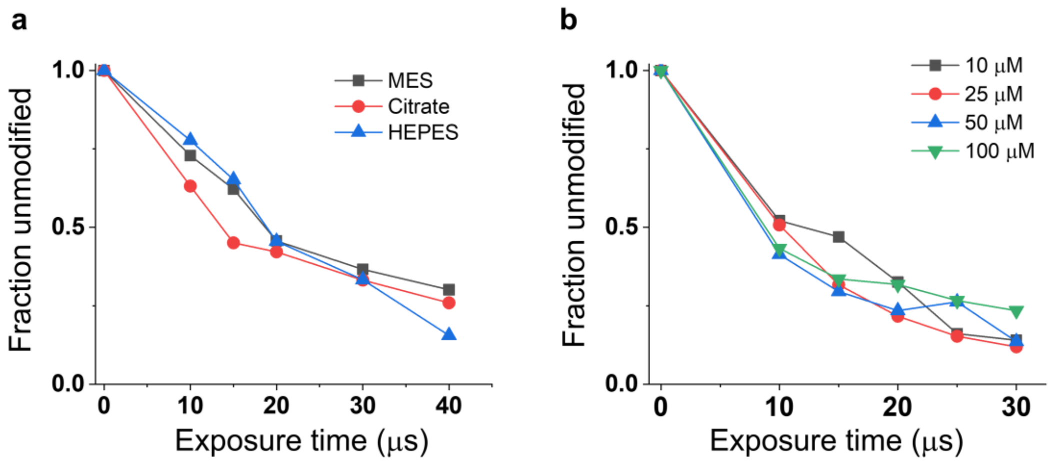

Supplementary Figure S20. Alexa dose-response plot obtained by manual Alexa fluorescence measurements using a standalone fluorescence spectrometer for the exposed 10 mM MES, Citrate and HEPES buffer solutions (a) and cyt c protein samples in 10 mM phosphate (b) at micro-focused high flux density beamline 17-BM of NSLS-II. The dose-response kinetic traces were insensitive to the scavenging properties of buffer and protein.

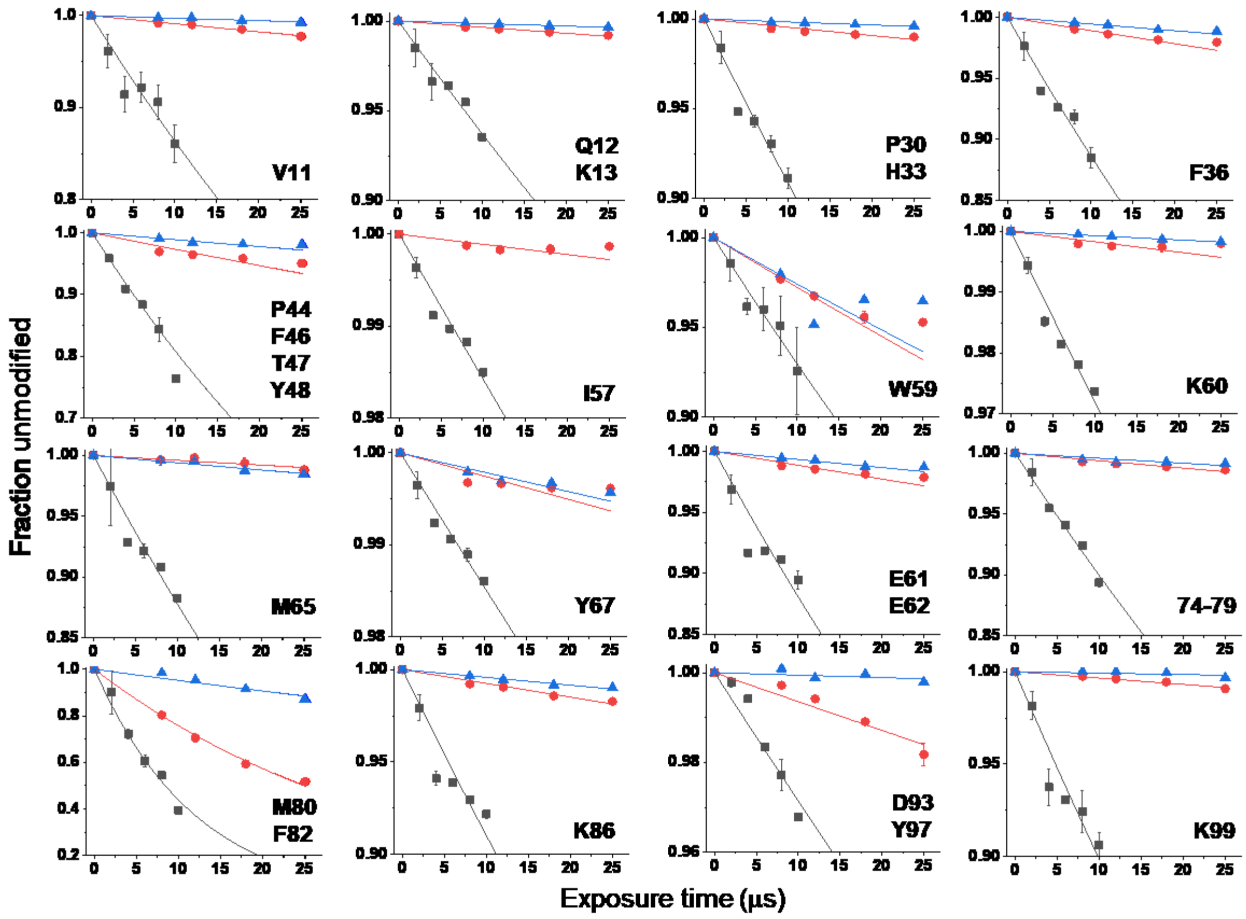

Supplementary Figure S21. Comparison of residue-specific dose-responses of cyt c in HEPES (blue), Tris (red) and phosphate (black). The exposure time range for the Tris and HEPES is exended to double digit microseconds using a 100 μm jet.

Supplementary Table 1. Experimental parameters for running liquid jet sample exposure and testing

**Light intensity-dependent PMT test**						
**Exposure time (μs)**	**Jet ID (μm)**	**Sample vol (μl)**	**Flow rate (ml/min)**	**Jet speed (m/s)**	**Total pump time (ms)**	**Exposure length (mm)**
7.8	50	25	3.02	25.6	0.497	0.2
10	50	25	2.36	20	0.637	0.2
15	50	25	1.57	13.3	0.955	0.2
20	50	25	1.18	10	1.27	0.2
30	50	25	0.785	6.7	1.91	0.2
7.8	100	25	1.51	12.8	0.993	0.1
10	100	25	1.18	10	1.27	0.1
15	100	25	0.785	6.7	1.91	0.1
20	100	25	0.589	5	2.55	0.1
30	100	25	0.393	3.3	3.82	0.1
**Single-digit microsecond exposure range for protein samples at NSLS-II 17-BM**						
2	50	50	5.89	50	0.509	0.1
4	50	50	2.95	25	1.02	0.1
6	50	50	1.96	16.7	1.53	0.1
8	50	50	1.47	12.5	2.04	0.1
10	50	50	1.18	10	2.55	0.1
2	50	25	5.89	50	0.2545	0.1
4	50	25	2.95	25	0.51	0.1
6	50	25	1.96	16.7	0.765	0.1
8	50	25	1.47	12.5	1.02	0.1
10	50	25	1.18	10	1.275	0.1
**Double-digit microsecond exposure range for protein samples at NSLS-II 17-BM**						
8	100	50	5.89	12.5	0.509	0.1
12	100	50	3.93	8.3	0.764	0.1
18	100	50	2.62	5.6	1.15	0.1
25	100	50	1.88	4	1.59	0.1
8	100	25	5.89	12.5	0.255	0.1
12	100	25	3.93	8.3	0.382	0.1
18	100	25	2.62	5.6	0.573	0.1
25	100	25	1.88	4	0.796	0.1
**Alexa dose-response for buffer samples at NSLS-II 17-BM**						
10	100	50	4.71	10	0.637	0.1
15	100	50	3.14	6.7	0.955	0.1
20	100	50	2.36	5	1.27	0.1
30	100	50	1.57	3.3	1.91	0.1
40	100	50	1.18	2.5	2.55	0.1

**Table S1 continued.**						
10	100	25	4.71	10	0.318	0.1
15	100	25	3.14	6.7	0.477	0.1
20	100	25	2.36	5	0.637	0.1
30	100	25	1.57	3.3	0.955	0.1
40	100	25	1.18	2.5	1.27	0.1
**Double-digit microsecond exposure for protein and buffer samples at ALS 5.3.1**						
10	75	50	3.18	12	0.943	0.12
15	75	50	2.12	8	1.41	0.12
20	75	50	1.59	6	1.89	0.12
25	75	50	1.27	4.8	2.36	0.12
30	75	50	1.06	4	2.83	0.12

Supplementary Table 2. Hydroxyl radical reactivity rate constants for Alexa 488 in the presence of X-ray beam attenuation by aluminum

Al plate thickness (μm)	*k* sec^{-1}
0	338626.83 ± 18772.34
25.4	226539.96 ± 8070.18
50.8	168495.02 ± 7367.79
76.2	122023.82 ± 7031.56
101.6	102300.56 ± 7111.92
127	76158.69 ± 5176.48
152.4	67370.91 ± 4634.27
203.2	52900.44 ± 4190.04
254	41497.13 ± 3145.22
381	26242.92 ± 1735.60
508	17409.70 ± 961.17
635	12601.17 ± 462.02
762	8546.40 ± 129.20

Supplementary Table 3: Rate constants of hydroxyl radical modification for cyt *c* prepared in 10 mM phosphate pH 7, 10 mM Tris pH 7, and HEPES pH 7

Sequence of the trypsin fragments[a]	Sites of modification[b]	Hydroxyl radical reactivity rate k (s^{-1})[c]		
		Phosphate	Tris	HEPES
[9]IFVQK[13]	V11	14.67 ± 1.2	0.89 ± 0.03	0.28 ± 0.02
	Q12, K13	6.48 ± 0.3	0.34 ± 0.02	0.14 ± 0.003
[28]TGPNLHGLFGR[38]	P30, H33	9.6 ± 0.5	0.5 ± 0.04	0.2 ± 0.01
	F36	12.9 ± 0.6	1.1 ± 0.04	0.6 ± 0.01
[40]TGQAPGFTYTDANK[53]	P44, F46, T47, Y48	21.4 ± 0.5	2.7 ± 0.3	1.1 ± 0.07
[56]GITWK[60]	I57	1.6 ± 0.1	0.1 ± 11.3	-
	W59	7.3 ± 0.4	2.8 ± 0.05	2.6 ± 0.5
	K60	2.8 ± 0.1	0.17 ± 0.02	0.07 ± 0.002
[61]EETLMEYLENPK[72]	M65	1.3 ± 0..8	0.4 ± 0.06	0.6 ± 0.05
	Y67	1.5 ± .07	0.25 ± 0.04	0.21 ± 0.02
	E61,E62,	12.7 ± 1.2	1.2 ± 0.09	0.7 ± 0.02
[74]YIPGTK[79]	Mixed[d]	10.5 ± 0.3	0.61 ± 0.04	0.41 ± 0.05
[80]MIFAGIK[86]	M80, F82	82.4 ± 4.2	27.8 ± 0.6	4.9 ± 0.5
	K86	9.4 ± 0.8	0.75 ± .04	0.4 ± 0.02
[92]EDLIAYLK[99]	D93, Y97	2.9 ± 0.2	0.64 ± 0.06	0.05 ± 0.03
	K99	10.7 ± 0.8	0.34 ± 0.01	0.08 ± 0.03

[a] sequences of tryptic fragments

[b] modified residues, which were identified and confirmed by LCMS as previously described

[c] hydroxyl radical rate constants were estimated by employing a first-order exponential fit of the dose-response plot of overall peptide modification as described in experimental procedures and Figure S5. The modified peptide fragments were eluted as a single peak or multiple peaks. The modified peak areas were extracted individually but summed together to calculate the total modification of the respective peptide.

Supplementary Table 4: Rate constants of hydroxyl radical modifications for phycobilisome subunits prepared in 800 mM phosphate pH 7

Subunit & modified residue[a]	Hydroxyl radical reactivity rate k (s^{-1})[b]
ApcA, M85	0.75 ± 0.1
ApcA, M141	0.41 ± 0.02
ApcA, Y152	1.54 ± 0.2
ApcB, M1	0.72 ± 0.04
ApcB, M96	0.57 ± 0.2
ApcB, M149	0.37 ± 0.08
ApcD, L89	0.54 ± 0.06
ApcD, Y88	1.63 ± 0.3
ApcE, M269	2.33 ± 0.3
ApcE, M667	0.65 ± 0.05
ApcF, M86	0.75 ± 0.1
CpcA, F18	0.24 ± 0.03
CpcA, P126	0.08 ± 0.01
CpcA, D108	0.17 ± 0.03
CpcB, M1	1.58 ± 0.1
CpcB, N54	0.05 ± 0.01
CpcB, M85	1.93 ± 0.1
CpcC, N64	0.7 ± 01

[a] modified residues, which were identified and confirmed by LCMS

[b] hydroxyl radical rate constants were estimated by employing a first-order exponential fit of the dose-response plot as described in the experimental procedures and shown in the main text Figure 5.

Supplementary References

1. Garg, B., & Sharma, G.K. (2016). A quality-aware Energy-scalable Gaussian Smoothing Filter for image processing applications. Microprocessors and Microsystems, 45(A), 1-9.

2. Khan, Y., Ahmed, F., & Khan S. (2013). Situation recognition using image moments and recurrent neural networks. Neural Computing and Applications, 24, 1519-1529.

3. Belkasim S.O., Shridhar M., & Ahmadi M. (1991). Pattern recognition with moment invariants. A comparative study and new Results. Pattern Recognition 24(12):1117–1138

4. Dudani S.A., Breeding K.J., & McGhee R.B. (1977) Aircraft identification by moment invariants. IEEE Transactions on Computers, C-26(1):39–46

5. Canny Edge Detection. (2013). Retrieved from https://opencv-python-tutroals.readthedocs.io/en/latest/py_tutorials/py_imgproc/py_canny/py_canny.html

6. Sahir, S. (2019). Canny Edge Detection Step by Step in Python — Computer Vision. Retrieved from https://towardsdatascience.com/canny-edge-detection-step-by-step-in-python-computer-vision-b49c3a2d8123

7. Gupta, S., Chen, Y., Petzold, C. J., DePonte, D. P. & Ralston, C. Y. Development of Container Free Sample Exposure for Synchrotron X-ray Footprinting. Anal Chem 92, 1565-1573, doi:10.1021/acs.analchem.9b04849 (2020).
