# Supplementary material for: An automated liquid jet for fluorescence dosimetry and microsecond radiolytic labeling of proteins
Source: Commun Biol. 2022 Aug 25;5:866. doi: 10.1038/s42003-022-03775-1 (PMC9411504; doi:10.1038/s42003-022-03775-1)
Supplement: Supplementary file 2 — Description of Additional Supplementary Files [file 42003_2022_3775_MOESM2_ESM.pdf]

Description of Additional Supplementary Files

File name: Supplementary Data 1
Description: Numerical source data for figures 2, 3, and 5.

File name: Supplementary Data 2
Description: Numerical source data for figure 4.
